# Microemulsions as Lipid Nanosystems Loaded into Thermoresponsive In Situ Microgels for Local Ocular Delivery of Prednisolone

**DOI:** 10.3390/pharmaceutics14091975

**Published:** 2022-09-19

**Authors:** Rania Hamed, Amani D. Abu Kwiak, Yasmeen Al-Adhami, Alaa M. Hammad, Rana Obaidat, Osama H. Abusara, Rana Abu Huwaij

**Affiliations:** 1 Department of Pharmacy, Faculty of Pharmacy, Al-Zaytoonah University of Jordan, Amman 11733, Jordan; 2 Department of Pharmacy, Faculty of Pharmacy, Zarqa University, Zarqa 13110, Jordan; 3 Department of Pharmaceutical Technology, Faculty of Pharmacy, Jordan University of Science and Technology, Irbid 22110, Jordan; 4 Pharmacological and Diagnostic Research Center, Faculty of Pharmacy, Al-Ahliyya Amman University, Al-Salt 19328, Jordan

**Keywords:** Pluronic^®^ F127, kolliphor^®^ P188 (F68), prednisolone, in situ thermoresponsive microgel, rheological properties, mucoadhesion, local ocular delivery, microemulsion, eye irritation, mixed polymeric micelles

## Abstract

This study aimed to develop and evaluate thermoresponsive in situ microgels for the local ocular delivery of prednisolone (PRD) (PRD microgels) to improve drug bioavailability and prolong ocular drug residence time. Lipid nanosystems of PRD microemulsions (PRD-MEs) were prepared and evaluated at a drug concentration of 0.25–0.75%. PRD microgels were prepared by incorporating PRD-MEs into 10 and 12% Pluronic^®^ F127 (F127) or combinations of 12% F127 and 1–10% Kolliphor^®^P188 (F68). PRD microgels were characterized for physicochemical, rheological, and mucoadhesive properties, eye irritation, and stability. Results showed that PRD-MEs were clear, miscible, thermodynamically stable, and spherical with droplet size (16.4 ± 2.2 nm), polydispersity index (0.24 ± 0.01), and zeta potential (−21.03 ± 1.24 mV). The PRD microgels were clear with pH (5.37–5.81), surface tension (30.96–38.90 mN/m), size, and zeta potential of mixed polymeric micelles (20.1–23.9 nm and −1.34 to −10.25 mV, respectively), phase transition temperature (25.3–36 °C), and gelation time (1.44–2.47 min). The FTIR spectra revealed chemical compatibility between PRD and microgel components. PRD microgels showed pseudoplastic flow, viscoelastic and mucoadhesive properties, absence of eye irritation, and drug content (99.3 to 106.3%) with a sustained drug release for 16–24 h. Microgels were physicochemically and rheologically stable for three to six months. Therefore, PRD microgels possess potential vehicles for local ocular delivery.

## 1. Introduction

Ocular inflammatory disease (OID) may occur at any age in response to infections, allergies, immune-mediated inflammation, irritation, injury, or trauma of the eyes, eyelids, or surrounding tissues [1]. The symptoms of OID include redness, pain, edema, and reduced ocular motility, which are commonly treated with corticosteroids [2]. Due to its potential anti-inflammatory and immunosuppressive properties, prednisolone (PRD) is the most widely used corticosteroid in OID [3]. PRD potentially inhibits the production of prostaglandins and leukotrienes. Therefore, its local administration is preferable in topical diseases, where topical formulations can provide higher drug concentration at the administration site in a sustained-release manner [4]. PRD is a Biopharmaceutics Classification System class I or borderline class II. It is practically insoluble in water, where 1 g of PRD dissolves in >1000 mL [5].

Microemulsions are lipid nanosystems that have been widely used to improve the aqueous solubility of drugs [6]. Mainly, ocular microemulsions are intended to achieve a sustained drug release when applied to the cornea and attain a higher drug concentration by enhancing its penetration into the deeper layers of the eye and aqueous humor. This reduces the frequency of drug administration and side effects, elevates patient compliance, and increases ocular bioavailability compared to conventional eye formulations [7]. In addition, ocular microemulsions are easily sterilized and prepared [8].

The thermoresponsive in situ gels have been widely employed to sustain the ocular delivery of drugs and prolong ocular residence time [9]. These systems are transparent polymeric solutions that are liquids at storage conditions and convert into gels once inserted into the eye, owing to the phase transition properties of the thermoresponsive polymers [10]. Pluronics are one of the commonly used thermoresponsive polymers that are composed of a triblock copolymer that exhibits amphiphilic properties owing to the hydrophilic domains poly(ethylene oxide) (PEO) and the hydrophobic domains poly(propylene oxide) (PPO). The self-assembly of the amphiphilic polymer leads to the formation of unique polymeric micelles as a core–shell type [11]. The combination of Pluronics F68 and F127, which forms mixed polymeric micelles (MPM), is one of the ideal nanocarrier systems for poorly soluble drugs [12]. Additionally, the gelation of Pluronics varies based on the micelle structure, which is influenced by the Pluronic type, concentration, and temperature [13,14]. Moreover, the incorporation of microemulsions into thermoresponsive in situ systems has previously been used to sustain the ocular delivery of drugs, where systems convert into gels at a physiological temperature [15].

Therefore, the objective of this study was to develop and evaluate thermoresponsive in situ microgels for the ocular delivery of PRD to improve drug bioavailability and prolong ocular drug residence time. Microgels were prepared by incorporating the lipid nanosystems oil-in-water (O/W) microemulsion, loaded with PRD, into the thermoresponsive Pluronic F68 and F127 hydrogels. The term microgel has been used in our previous studies to refer to a drug delivery system prepared by combining an O/W microemulsion, loaded with a drug, and the in situ thermoresponsive hydrogels to regulate drug release upon responding to the physiological temperature at the site of action [13,14]. PRD is a poorly soluble drug [5]. Therefore, a combination of O/W microemulsion and MPM of F68 and F127 were used to enhance the solubility of PRD. In addition, the thermoresponsive properties of microgels, which gel at the physiological temperature of the eye, controlling the release of drug at the site of action.

Initially, a series of O/W microemulsions were developed at a PRD concentration of 0.25–0.75% (PRD microemulsions, PRD-MEs) using the spontaneous emulsification method. PRD-MEs were characterized in terms of mean droplet size (MDS), polydispersity index (PDI), zeta potential (ZP), thermodynamic stability, and droplet morphology. To prepare PRD microgels, PRD-MEs were incorporated into either 10 and 12% Pluronic^®^ F127 (Sigma-Aldrich, St. Louis, MO, USA) (F127) or combinations of 12% F127 and 1–10% Kolliphor^®^ (Sigma-Aldrich, St. Louis, MO, USA) P188 (F68). PRD microgels were characterized for clarity, pH, surface tension, phase-transition temperature (T_sol__→gel_), and gelation time (T_gel_). FTIR spectroscopy was performed to evaluate drug excipients compatibility. Additionally, the thermal behavior, rheological (viscosity and viscoelasticity), and mucoadhesive (microgel–mucin interaction) properties, eye irritation, drug content, and rate of drug release of PRD microgels were assessed. An HPLC method was used to determine the amount of PRD in the tested samples. The thermal and rheological stability of PRD microgels was evaluated after three and six months, respectively, at various storage conditions.

## 2. Materials and Methods

### 2.1. Materials

Prednisolone (PRD) was kindly donated by Hikma Pharmaceuticals (Amman, Jordan). Pluronic^®^ F127 (F127) and Kolliphor^®^ P188 (F68) were obtained from Sigma-Aldrich (St. Louis, MO, USA). Propylene glycol (PG) was obtained from Dow Chemicals Company (Midland, MI, USA), polyethylene glycol 400 (PEG 400) from INEOS AG (Rolle, Switzerland), ethanol (99.9%) from Solvochem (Rotterdam, The Netherlands), Tween^®^ 20 (TW-20) from Tedia (Fairfield, OH, USA), and oleic acid (OA) from Fisher (Shanghai, China). Isopropyl myristate (IPM) was a gift from Evonik (Essen, Germany), and caprylic/Capric Triglycerides (Labrafac^®^ CC, LC, Lyon, France), diethylene glycol monoethyl ether (Transcutol P^®^, TC-P, Lyon, France), and caprylocaproyl polyoxyl-8 glycerides NF (Labrasol^®^, LB, Lyon, France) were gifts from Gattefossé (Saint Priest, France). Mucin from porcine stomach (Type III, partially purified mucin) was obtained from Sigma (St. Louis, MO, USA), acetonitrile (HPLC grade) from Fisher Chemical (Loughborough, UK), tetrahydrofuran (THF) from Sigma-Aldrich (Lyon, France), and cellulose dialysis membrane (MW cut-off 12,000–14,000 Da) from Medicell International Ltd. (London, UK). All chemicals were of analytical grade and used as received.

### 2.2. Preparation of Prednisolone Microemulsions (PRD-MEs)

#### 2.2.1. Solubility of Prednisolone

The solubility of prednisolone (PRD) was assessed in different oils (OA, IPM, and LC), surfactants (TW-20 and LB), cosurfactants (ethanol, PG, PEG 400, and TC-P), and water, as previously described [16]. Briefly, PRD was added in excess to 2 mL of oils, surfactants, cosurfactants, and water in screwed test tubes. Samples were vortexed and kept to dissolve in an isothermal shaker maintained at 37 ± 1 °C for 24 h to equilibrate. Then, they were removed from the shaker, centrifuged for 15 min at 3500 rpm, and the supernatant was diluted suitably with methanol. The concentration of PRD was determined using UV spectrophotometry (Varian, Cary UV/VIS spectrophotometer) at 240 nm using a calibration curve created by preparing a set of PRD standard solutions.

#### 2.2.2. Preparation of Pseudoternary Systems

A series of pseudoternary systems consisting of oil, a combination of surfactant and cosurfactant (S_mix_), and water as an aqueous phase was prepared by the aqueous titration method. S_mix_ combinations were prepared by mixing TW-20 or LB with PG or ethanol at different ratios (1:1, 1:2, and 2:1 *w*/*w*). The S_mix_ mixtures were tested visually for miscibility and transparency (clarity). S_mix_ mixtures that were immiscible and turbid were excluded from further studies. For preparing the pseudoternary systems, oil was initially added to the S_mix_, mixed to get a clear mixture, and then the aqueous phase was slowly added to the oil/S_mix_ mixture. Samples were vortexed and left for an equilibration time of 1–2 min after each addition of water. The pseudoternary systems were visually assessed for appearance, fluidity, clarity, miscibility (no phase separation), and transparency [17]. Systems that were clear, able to flow easily, and miscible passed the visual testing, hence subjected to further studies, whereas systems that showed turbidity, poor flowability, and immiscibility (phase separation) did not pass the visual testing and were excluded from the study. Table 1 illustrates the composition of the pseudoternary systems that passed or failed the visual test.

#### 2.2.3. Preparation of PRD-MEs

To prepare PRD-MEs, PRD was loaded into the pseudoternary systems that passed the visual test. PRD at concentrations of 0.25, 0.5, 0.75, and 1% was dissolved in OA and selected systems of S_mix_. Then, the aqueous phase of either distilled water or 40% F68 was added to the prepared mixture using the aqueous titration method (Table 2).

### 2.3. Characterization of PRD-MEs

#### 2.3.1. Droplet Size, Polydispersity Index, and Zeta Potential

The mean droplet size (MDS), polydispersity index (PDI), and zeta potential (ZP) of the selected PRD-MEs were determined at 25 °C by a nanosizer (Nicomp Nano Z3000, Santa Barbara, CA, USA). Measurements were determined initially and every week for up to one month. Dilution for PRD-MEs with distilled water at a ratio of 0.5:4.5 (*v*/*v*) was required for the measurements. Measurements were assessed in triplicate.

#### 2.3.2. Thermodynamic STABILITY Studies

The thermodynamic stability of PRD-MEs was assessed by centrifugation, heating/cooling cycles, and freeze/thaw cycles, as described [18]. Initially, microemulsions were centrifuged at 3500 rpm for 15 min. If no phase separation was observed, PRD-MEs were subjected to six cycles of heating (45 °C) and cooling (4 °C) for 48 h at each temperature. If PRD-MEs remained clear, they were subjected to three cycles of freezing (−21 °C) and thawing (25 °C) for 48 h at each temperature.

#### 2.3.3. Transmission Electron Microscope (TEM)

A transmission electron microscope (TEM, FEI Morgani 268, operating voltage of 60 kV, Brielle, The Netherlands) connected to a Mega View II digital camera was performed to assess the morphology and particle size of the selected PRD-MEs.

Initially, one drop of PRD-ME was diluted with distilled water at a ratio of 1:2 *v*/*v*, then placed on a carbon-coated copper grid and allowed to dry before imaging.

### 2.4. Preparation of PRD Microgels

#### 2.4.1. Preparation of F127/F68 Hydrogels

F127/F68 hydrogels were prepared using the cold method described [13]. Initially, hydrogels were prepared from F127 at concentrations of 10 and 12% or from combinations of F127 at 12% and F68 at concentrations of 1, 2.5, 5, 7.5, and 10%. F127 and F68 were added to the cold water with continuous stirring for 6 h. Then, hydrogels were kept in the refrigerator overnight, giving appropriate time for complete polymer dissolution.

#### 2.4.2. Preparation of PRD Microgels

PRD microgels were prepared by combining F127 or F127/F68 hydrogels with PRD-MEs at PRD concentrations of 0.25, 0.5, and 0.75% (Table 3). For instance, to prepare 10 mL of M7 microgel (Table 3), which is composed of 12% F127 and 10% F68, weighed amounts of 1.2 g F127 and 1 g F68 were added to 6 mL of cold distilled water while placed in an ice bath with continuous stirring for 6 h and then kept in the refrigerator overnight for complete dissolution. Then, a volume of 2 mL RRD-ME was added to F127/F68 hydrogels by titration and placed in an ice bath with continuous stirring. Finally, samples were kept in the refrigerator overnight.

### 2.5. Characterization of PRD Microgels

#### 2.5.1. Clarity, pH, and Surface Tension

The clarity of PRD microgels was assessed by visual examination under black and white background as described [19]. Additionally, the pH of the microgels was measured using a pH meter (Mettler Toledo, Columbus, OH, USA). The surface tension measurements of microgels and water (used as a control) were carried out at room temperature (20–22 °C) using the Wilhelmy plate (Sigma 700/701 tensiometer, Biolion Scientific, Gothenburg, Sweden). Before each measurement, a 50 mL glass vessel used to hold the sample was perfectly cleaned with water and alcohol. Additionally, the plate was cleaned by flame and calibrated with deionized water. Then, the vessel was placed onto the tensiometer platform, and the plate was submerged and then slowly raised to form a meniscus of liquid and measure the surface tension. The surface tension measurements were assessed in triplicate.

#### 2.5.2. Size and Zeta Potential

The size and zeta potential (ZP) of MPM of M 2-7 microgels were determined at 25 °C by a nanosizer (Nicomp Nano Z3000, Santa Barbara, CA, USA). Dilution for PRD microgels with distilled water at a ratio of 0.5:4.5 (*v*/*v*) was required for the measurements. Measurements were assessed in triplicate.

#### 2.5.3. Sol–Gel Transition Temperature (T_sol→gel_) and Gelation Time (T_(gel)_)

The test tube inversion method was used to determine the sol–gel transition temperatures (T_sol→gel_) of PRD microgels over a temperature range of 10–50 °C as described [20]. The gelation time (T_(gel)_) was recorded when the microgel completely gelled upon turning over the tube. All measurements were assessed in triplicate.

#### 2.5.4. Physicochemical Characterization of PRD Microgels

##### Fourier Transform Infra-Red (FTIR)

The FTIR spectra were carried out for PDR, F127, F68, and PRD microgels, and their corresponding physical mixtures (PM) using FTIR spectroscopy (Shimadzu-840-os, Tokyo, Japan) over frequencies between 4000 and 400 cm⁻^1^. The PDR, F127, F68, and PM samples were grounded and prepared as potassium bromide disks, whereas the FTIR spectra of PRD microgels were performed using NaCl plate.

##### Differential Scanning Calorimetry (DSC)

The thermal transitions of F127, F68, PRD powder, PRD microgels, and their corresponding PM were determined using differential scanning calorimetry (DSC 204 F1 Phoenix, Netzsch, Germany). Accurately weighed samples (5 mg) were placed in aluminum pans and hermetically sealed. The DSC analysis was carried out at a temperature range of 10–300 °C and a heating rate of 5 °C/min.

#### 2.5.5. Rheological Studies

##### Viscosity Curves

The viscosity curves of PRD microgels were performed using a controlled-stress rheometer (CSR) (Anton Paar MCR 302, Graz, Austria) at 35 °C. A cone/plate system with a 1° cone angle and 25 mm plate diameter was used for viscosity measurement over shear rates between 0.1 and 100 s^−1^. A 0.5 g of PRD microgel was applied onto the plate, then left to rest for ~1 min before measurement.

##### Mathematical Modeling of Viscosity Data

The viscosity curves of PRD microgels were fitted into Carreau–Yasuda and Casson models [21]. The Carreau–Yasuda model (Equation (1)) was used to investigate the pseudoplastic fluids:

(1) $$\frac{\eta -\eta \infty }{\eta {}^{\circ}-\eta \infty }={\left\{\;1+\left(\lambda \gamma \right)a\right\}}^{\left(n-1\right)/a}$$

where η: viscosity, η^°^: zero viscosity, η∞: infinite viscosity, γ: shear rate, and λ, α, n are the shape parameters. The Casson model (Equation (2)) was fitted to estimate the yield stress:

(2) $$\tau^{1/2}-\tau {ο}^{1/2}=\eta \infty^{1/2\;}\gamma^{1/2}$$

where τ: shear stress and το: yield stress, η∞: infinite shear viscosity, and γ: shear rate.

##### Strain–Sweep Studies

Strain–sweep studies were performed using the CSR at 35 °C to investigate the linear viscoelastic region (LVR) for PRD microgels at strain values between 0.01 and 100% as described [22]. Briefly, a 0.5 g microgel sample was loaded onto the CSR plate and left to relax for 1 min. The cone was lowered, and the gap between the cone and plate was adjusted to 0.05 mm. Throughout the test, the cone was oscillated at a constant frequency of 6.28 rad/s. The strain–sweep studies were assessed in triplicate for each microgel.

##### Frequency–Sweep Studies

The frequency–sweep studies for PRD microgels were performed over frequencies between 0.1 and 100 rad/s at 35 °C. Studies were conducted at constant strain, carefully chosen from the strain–sweep studies for each microgel [22]. The frequency-dependent elastic (G′) and viscous (G″) moduli were assessed for at least six replicates for each microgel.

##### Temperature–Sweep Test

The temperature–sweep test was performed to assess the T_sol→gel_ and curing temperature (T-_Curing_) of PRD microgels during the phase transition as described [23]. The measurements were carried out at a constant shear rate of 50 s^−1^, temperature range of 10–50 °C, and heating rate of 5 °C/min.

### 2.6. Mucoadhesion Test

The mucoadhesive properties of PRD microgels were assessed using rheological synergism resulting from mixing PRD microgels with different concentrations of mucin dispersions as described [24]. Samples of mucin dispersions and PRD microgels with the addition of mucin (mucin/PRD microgel mixtures) were subjected to frequency–sweep tests at 35 °C over frequencies between 0.1 and100 rad/s and at a constant strain selected in the LVR as described in Section Strain–Sweep Studies.

#### 2.6.1. Preparation of Mucin Dispersions

Mucin dispersions were prepared at a concentration range of 2–5%. A proper amount of mucin was soaked in distilled water at 4 °C for 24 h. Then, the dispersion was gently stirred for sufficient time at room temperature to get a homogenous dispersion as described [24].

#### 2.6.2. Preparation of Mucin/PRD Microgel Mixtures

To obtain homogenous mucin/PRD microgel mixtures, mucin dispersion was mixed with PRD microgel using a high-performance digital laboratory stirrer (IKA^®^ Eurostar power control visc 6000, GmbH, Staufen im Breisgau, Germany). Mixtures were stored at 4 °C for no longer than 24 h. The mucoadhesive interaction parameter (ΔG′) was measured by substracting the elastic modulus of the mucin/PRD microgel mixture (G′ _mixture_) from the summation of their elastic moduli (G′ _microgel_ and G′ _mucin_) (Equation (3)) [25].

ΔG′ = G′ _mixture_ − (G′ _microgel_ + G′ _mucin_) (3)

ΔG′ was used as an indicator for rheological synergism where positive ΔG′ indicates mucoadhesive interaction between mucins and PRD microgels, whereas negative ΔG′ indicates the absence of mucoadhesive interaction between mucins and PRD microgels [26]. However, the elastic modulus G′ of mucin dispersions (2–5%) was negligibly small, both in water and in simulated tear fluid (STF), in agreement with that reported previously [24]. Therefore, Equation (3), used to calculate ΔG′, was simplified to Equation (4) as reported [27,28]:

ΔG′ = G′ _mixture_ − G′ _microgel_ (4)

### 2.7. Eye Irritation Test

A pilot study on the eyes of two American white rabbits was conducted to provide a preliminary investigation on the eye irritation of the selected PRD microgels. The Research Ethics Committee at Al-Zaytoonah University of Jordan has reviewed and approved the application to perform the in vivo study on the rabbit eyes under IRB number 22/04/2021–2022. About 50 µL microgels were instilled into the lower conjunctival sac of the lower eyelid of the rabbit’s right eye. The left eye of the same rabbit was left as a control, where 50 µL of a blank microgel (microgel prepared without PRD) was instilled. Each eye was observed and investigated in terms of ocular irritation parameters. These include conjunctival chemosis, swelling, redness, and the presence of any discharge. It was graded on a scale range from 0 to 3, 0 to 4, 0 to 3, and 0 to 3, respectively, for each sign, following the modified Draize test [29]. After administration, an eye examination was carried out at designed intervals (0, 10, and 30 min, and 1, 2, 3, 4, 6, and 24 h). Based on the observations, the score was given for each eye on a scale from 0 (no irritation) to 4 (highest irritation) [30]. The overall eye score was calculated by summing up the total evaluation grades at each time interval. Each eye score of 2 or 3 in any category or total grades higher than 4 over observation time was considered a significant irritant [31].

### 2.8. High-Performance Liquid Chromatography

High-performance liquid chromatography (HPLC, Thermo Scientific™ Dionex™ UltiMate™ 3000 U/HPLC, Chromeleon 7.2 software, Germering, Germany) was used to quantify PRD as described [32]. The chromatographic conditions were: mobile phase (water:THF: acetonitrile, 75:15:10, *v*/*v*), flow rate: 1 mL/min, column: RP-C18 (150 × 4.6 mm, 5 µm (Universil), and injection volume: 20 μL. The mobile phase was passed through a 0.45 µm regenerated cellulose membrane and then degassed. The UV detection wavelength for PRD was set at 254 nm. First, a stock solution was prepared by dissolving 25 mg PRD in 50 mL mobile phase and sonicated to aid complete dissolution. Then, a standard solution was prepared by transferring 5 mL of the stock solution into another 50 mL mobile phase. The standard solution was then serially diluted to prepare a calibration curve of PRD. Finally, PRD was eluted at 5.5 min.

### 2.9. Assay Test

The PRD content in microgels was assessed using the HPLC analytical method. One gram of microgel was placed in a 25 mL volumetric flask. The mobile phase was used as a diluent. Samples were placed in the sonicator for 15 min and centrifuged for 10 min at 3500 rpm. The supernatant was diluted if needed, filtered, and analyzed by HPLC. The assay test was assessed in triplicate for each microgel.

### 2.10. In Vitro Release Studies

Sink conditions were maintained to ensure the solubility of PRD in the release medium. The in vitro release studies were performed using automated vertical diffusion cells (Hanson, SC, USA) to evaluate the diffusion of PRD at 35 °C. The cells had a receptor compartment volume of 9 mL and an orifice surface area of 1.76 cm^2^. A simulated tear fluid (STF, pH 7.4) composed of the electrolytes of tear fluid was prepared by dissolving 0.22 g NaHCO, 0.68 g NaCl, 0.01 g CaCl_2_, and 0.14 g KCl in 100 mL ultrapure water [33]. Before use, the cellulose membrane was soaked in STF overnight. The receptor chamber was filled with STF and methanol (70:30 *v*/*v*). TW-20 (5%) was added to the release medium to ensure sink condition. One gram of PRD microgel was placed on the cellulose membrane. The membrane was fitted between the donor and receptor compartments. At predetermined time intervals (0.15, 1, 2, 4, 8, 12, 16, 20, and 24 h), 1.2 mL was withdrawn from each cell to determine the drug content using the HPLC. Additionally, a fresh release medium of 1.2 mL was added to the receptor compartment to replace the withdrawn samples’ volume and maintain sink conditions.

### 2.11. Mechanism of Release

To determine the mechanism of the drug release, mathematical fitting models and linearization of the release were carried out to analyze the release data for PRD microgels. The kinetics of drug release data were determined using zero-order, first-order, Higuchi, and Korsmeyer–Peppas models.

### 2.12. Stability Studies of PRD Microgels

#### 2.12.1. Thermal Stability

The selected PRD microgels were subjected to thermal stability studies at room temperature (20–22 °C), 30 °C/65% relative humidity (RH), and 40 °C/75% RH. Samples were withdrawn every month for up to three months and were assessed for appearance and drug content as described in Section 2.9. In addition, the size and ZP of MPM were assessed after three months of storage at room temperature using the nanosizer. Finally, the value of each observation was compared with its corresponding initial data.

#### 2.12.2. Rheological Stability

To assess the effect of time on the rheological properties of the selected PRD microgels, the T_sol→gel_, T_(gel)_, viscosity, and viscoelastic properties were assessed after six months of storage at room temperature as described in Section 2.5.3, Section Viscosity Curves, and Section Frequency–Sweep Studies, respectively.

### 2.13. Statistical Analysis

One-way ANOVA followed by Tukey’s multiple comparisons was used to investigate the statistical significance of the solubility of PRD in oils and cosurfactants. In contrast, an unpaired t-test was used to investigate the statistical significance of the solubility of PRD in surfactants. All statistical analyses were performed using Prism-GraphPad 9.1 (GraphPad Software, Inc., San Diego, CA, USA) and were based on a *p* < 0.05 level of significance.

## 3. Results and Discussion

### 3.1. PRD-MEs

#### 3.1.1. Selection of PRD-MEs Components

**Figure 1:**
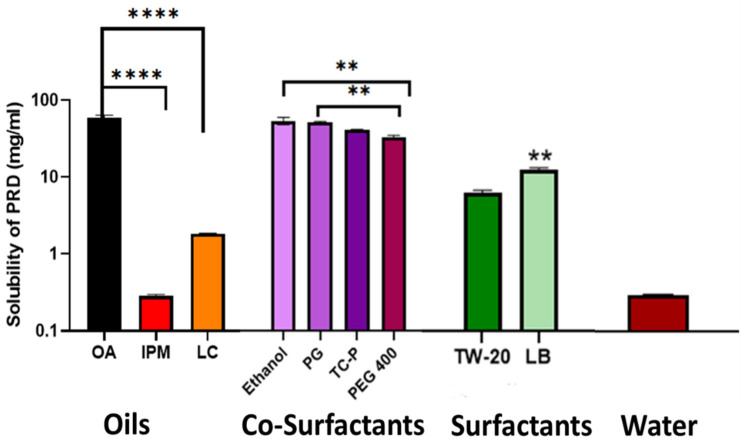
Solubility of PRD in oils (OA: oleic acid; IPM: isopropyl myristate; LC: Labrafac^®^ CC), surfactants (PG: propylene glycol; TC-P: Transcutol^®^ P; PEG 400: polyethylene glycol), cosurfactants (TW-20: Tween^®^ 20; LB: Labrasol^®^), and water. Data are presented as mean ± SD (*n* = 4). Significant results are marked with asterisks with *p <* 0.01 is given two asterisks and *p* < 0.0001 is given four asterisks.

PRD-MEs components were chosen based on the maximum solubility of PRD in oils, surfactants, cosurfactants, and water [16] (Figure 1). The solubility of PRD in OA was the highest compared to the other two oils (IPM and LC) (59.79 ± 7.01 mg/mL vs. 0.29 ± 0.02 and 1.85 ± 0.02 mg/mL, respectively) (*p* = 0.0001). Therefore, OA was selected as the oil phase for PRD-MEs. Additionally, there was no significant difference in PRD solubility between ethanol and PG (90.69 ± 12.31 and 88.30 ± 2.72 mg/mL, respectively) (*p* > 0.05), and both were higher than the solubility of PRD in TC-P and PEG 400 (75.46 ± 0.51 and 63.57 ± 4.90 mg/mL, respectively) (*p* = 0.004). Although the solubility of PRD in LB was higher than that in TW-20 (15.56 ± 0.90 vs. 10.73 ± 0.57 mg/mL, respectively) (*p* = 0.0013), TW-20 was also used in the pseudoternary phases of PRD-MEs. This is because TW-20 has been widely investigated as a surfactant in our studies [14,34,35,36]. Therefore, OA, ethanol, PG, TW-20, and LB were selected to prepare the pseudoternary phases of PRD-MEs. Furthermore, PRD solubility in water was very low at 0.33 ± 0.01 mg/mL.

#### 3.1.2. Pseudoternary Systems

Miscible and clear pseudoternary systems (S 1-6) were obtained with 10% *w*/*w* OA, 55% *w*/*w* S_mix_ of (TW-20/ethanol), and (TW-20/PG) at 1:1, 1:2, and 2:1 *w*/*w* ratios, and 35% *w*/*w* water. The rationale for choosing the ratio of the three phases (oil, S_mix_, and water, 10:55:35) was based on our previous studies, which showed that the ratios of oil, S_mix_, and water were 5–15, 50–60, and 30–40%, respectively [13,14,16,22,36,37]. Immiscible and turbid mixtures were detected when LB was used as a surfactant (S 7-12). Thus, they were excluded from further studies (Table 1).

#### 3.1.3. Preparation of PRD-MEs

PRD-MEs 1-6, prepared with the highest concentration of PRD (1%), showed drug precipitation (Table 2). To overcome this issue, F68, at a concentration of 40%, was added to the aqueous phase in order to increase the solubility of PRD and prevent its precipitation. Pham et al. [38] applied a similar approach by preparing an O/W microemulsion of PRD using OA as an oil phase and TW-20 and F68 as surfactants at different ratios. As a result, PRD-ME 7, prepared with 0.25% PRD and 35% *w*/*w* of 40% F68, was clear, showing no drug precipitation. Furthermore, the gradual increase in the concentration of PRD from 0.5 to 0.75% resulted in clear microemulsions with no drug precipitation (PRD-MEs 8-9). Adding F68 did not further solubilize PRD at its highest concentration (1%), resulting in drug precipitation. Therefore, PRD-MEs 1-6 and PRD-ME 10 were excluded from further studies, whereas PRD-MEs 7-9, which remained clear, were used to prepare PRD microgels. Finally, the pseudoternary systems prepared from S_mix_ TW-20: PG at different ratios (1:1, 1:2, and 2:1) passed the visual test, as illustrated in Table 1. However, these systems exhibited poor flowability, opposing one of the major criteria of microemulsions [22]. Additionally, when PRD was loaded into these systems to prepare PRD-MEs, the drug precipitated at various concentrations (0.25–1%). Thus, pseudoternary systems of TW-20: PG were excluded from the study.

### 3.2. Characterization of PRD-MEs

**Figure 2:**
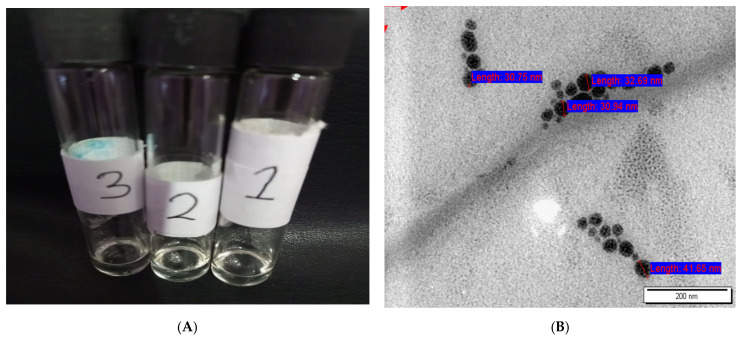
(**A**) Three samples (1, 2, and 3) of prednisolone microemulsion 7 (PRD-ME 7) after being subjected to thermodynamic stability studies showing no precipitation or phase separation and (**B**) Transmission electron microscope (TEM) image of PRD-ME 7.

PRD-ME 7 (0.25% PRD) was selected for further characterization and used in the preparation of microgels. The MDS, PDI, and ZP of PRD-ME 7 were measured every week for up to one month (Table 4). The initial results showed that the MDS of PRD-ME 7 was 16.4 ± 2.2 nm. After one month, although the droplet size of PRD-ME 7 slightly increased, ranging between 18.7 ± 2.8 and 67.9 ± 1.9 nm, the size remained within the acceptable nanosized range of microemulsions (10 and 100 nm [39]). The initial PDI of PRD-ME 7 was 0.24 ± 0.01. The PDI values of PRD-ME 7 remained small after one month ranging between 0.30 ± 0.04 and 0.35 ± 0.01, confirming the homogeneity of PRD-ME 7. Barot et al. [40] reported that the small PDI value indicates that the microemulsion droplets are homogenous with a narrow distribution. The initial ZP of PRD-ME 7 was −21.03 ± 1.24 mV. The relatively low negative ZP of PRD-ME 7 is attributed to the presence of the two nonionic surfactants TW-20 and F68 [41,42]. Generally, microemulsions are considered relatively stable if ZP exceeds −30 to +30 mV, as coalescence can be strongly inhibited [43]. The ZP of PRD-ME 7 decreased slightly with time, ranging between −9.71 ± 0.60 and −14.16 ± 2.21 mV. The slight decrease in ZP over time might be related to the slight coalescence of oil droplets by van der Waals forces [44]. Altogether, no clear difference was observed in the measurements of MDS, PDI, and ZP of PRD-ME 7 at different time intervals for up to 1 month. Moreover, PRD-ME 7 was physically stable, showing no phase separation or turbidity after centrifugation and heating/cooling and freezing/thawing cycles tests (Figure 2A). The image of TEM (Figure 2B) illustrates that the droplets of PRD-ME were of a uniform spherical shape with a particle size range of 30.75–41.65 nm, within the acceptable nanosized range of microemulsions (10 and 100 nm [39]).

### 3.3. PRD Microgels

#### 3.3.1. Clarity, pH, and Surface Tension

The PRD microgels were prepared using the cold method, one of the preferred methods for preparing Pluronic hydrogels. This is because the cold method provides clear in situ gels without forming any polymer lumps that might be obtained when a hot process is applied [45,46]. PRD microgels M 1-13 were prepared by loading 2 mL of 0.25–0.75% PRD-MEs 7-9 into F127 and F127/F68 hydrogels. PRD microgels were characterized in terms of clarity, pH, and surface tension (Table 3). Clarity is an important criterion for ocular preparations [47]. This is because visible particles may cause ocular irritation [48]. M1 microgel (F127 10%) was not clear and became turbid upon the addition of PRD-ME 7 (0.25% PRD). Hence, it was excluded from further studies. Increasing the concentration of F127 to 12% in M2 microgel (0.25% PRD) resulted in sufficiently clear microgel (Clear+) due to the increase in the micellar solubilization of PRD [49]. Additionally, due to the combination effect of micellar solubilization of 12% F127 and 1–10% F68, the clarity of M 2-7 microgels (0.25% PRD) was further enhanced (Clear++) [50]. M 8-13 microgels, prepared at higher PRD concentrations (0.5 and 0.75%), were not clear, and the drug precipitated despite the presence of 12% F12 and 0–10% F68. Thus, M 8-13 microgels were excluded from further studies.

The pH of M 2-7 microgels was between 5.43 ± 0.06 and 5.76 ± 0.05. Ammar HO et al. [51] reported that ocular formulations with a pH value range of 3.5 to 8.5 could be applied safely onto the eye, where the buffering capacity of the tears can adjust the pH of the preparation upon administration to the physiological tear’s pH of 7.45 [52].

The surface tension of M 2-7 microgels ranged between 31.2 ± 0.1 and 37.0 ± 1.9 mN/m. The surface tension of microgels was lower than that of lachrymal fluid at 40–50 mN/m [53], due to the presence of surfactants (TW-20, F127, and F68). The low surface tension enabled microgels to spread onto the eye’s surface and interact with the corneal epithelium [51].

#### 3.3.2. Size and Zeta Potential

Mixed polymeric micelles (MPM) are self-assembled colloidal dispersions that have gained significant attention owing to their small size, high thermodynamic stability, drug loading, and improved entrapment efficacy [12,54]. MPM was formed by mixing the amphiphilic block copolymers F127 and F68, at various concentrations, with the PRD-MEs. The size and ZP of MPM of M 2-7 microgels were between 20.2 ± 0.1 and 23.0 ± 0.9 nm (Table 5), slightly higher than that obtained for PRD-ME 7 (16.4 ± 2.2 nm, Table 4). In contrast, the ZP of MPM of M 2-7 microgels slightly shifted towards lower negative values, between −2.13 ± 0.79 and −8.95 ± 1.36 mV. The low ZP values indicated that the MPM of microgels is stabilized by steric hindrance rather than electrostatic interaction, owing to the presence of the nonionic PEO blocks of Pluronics [55].

#### 3.3.3. T_sol-gel_ and T_(gel)_

The ocular preparations should exhibit T_sol→gel_ at temperatures close to the eye’s surface (32.8–35.4 °C) [56]. The thermoresponsive polymers F127 alone or in combination with various concentrations of F68 have been used by Mahboobian et al. [57] to achieve a phase transition behavior that converts the thermoresponsive in situ gels of acyclovir from sol state (liquid) at room temperature to gel state at cul-de-sac temperature (between 30 and 35 °C).

Initially, M1 and M2 microgels were prepared using F127 only at 10 and 12% concentrations, respectively. M1 (10% F127) showed a T_sol→gel_ of 32.0 ± 0.5 °C, whereas M2 (12% F127) showed a lower T_sol→gel_ of 26.0 ± 0.7 °C (Table 3). This indicates that the concentration of F127 played a significant role in determining the T_sol→gel_ of microgels, where increasing F127 concentration decreased the T_sol→gel_. This agrees with Wei et al. [58], who reported that F127-based ocular thermoresponsive gels with an F127 concentration of <15% did not gel. Meanwhile, increasing F127 concentration to 25% enhanced the gelling properties, facing difficulties when injecting these formulations into the eyes as they gelled at ambient temperature.

The use of F127 only in microgels could not provide sufficiently clear microgels and appropriate gelation temperatures for in situ ocular delivery systems. A recent study by Obaidat et al. [14] showed that combining F127 and F68 at specific concentrations modified the gelation temperatures of in situ periodontal delivery systems. Thus, F68 was added to modulate the gelation behavior of microgels and attain a T_sol→gel_ close to the temperature of the eye’s surface (32.8–35.4 °C) [56]. The effect of adding F68 on the T_sol→gel_ of PRD microgels is illustrated in Table 3. M 3-7 microgels, prepared with 12% F127 and 1–10% F68, underwent a phase transition at T_sol→gel_ between 30.0 ± 0.5 and 35.0 ± 1.0 °C. The T_sol→gel_ increased with an increase in F68 concentration up to 7.5% and then decreased at F68 concentration of 10%. This is because a higher F68 concentration changes the PEO/PPO ratio, which contributes to the gelation process; hence, gelation occurs at lower T_sol→gel_ [59].

The T_(gel)_ of PRD microgels was corroborated T_sol→gel_, where microgels with higher T_sol→gel_ required longer T_(gel)_ to turn into a gel state. For M 2-7 microgels, the T_(gel)_ ranged between 1.47 ± 0.03 and 2.35 ± 0.12 min (Table 3). Fathalla et al. [60] reported that the T_(gel)_ of the ocular in situ gels of L-carnosine, prepared from Poloxamer-N407, was less than 1 min. The variation in the T_(gel)_ between M 2-7 microgels and that reported by Fathalla et al. [60] can be attributed to the variation in the composition of the two in situ gels. Additionally, the T_(gel)_ of microgels increased with an increase in F68 concentration up to 7.5%, then decreased at 10% F68 concentration, consistent with the T_sol→gel_ of microgels_._ Therefore, M 2-7 microgels were chosen for further studies since they revealed an acceptable T_sol→gel_ and T_(gel)_ within the required range for ocular delivery.

### 3.4. Physicochemical Characterization

#### 3.4.1. Fourier-Transform Infrared Spectroscopy (FTIR)

**Figure 3:**
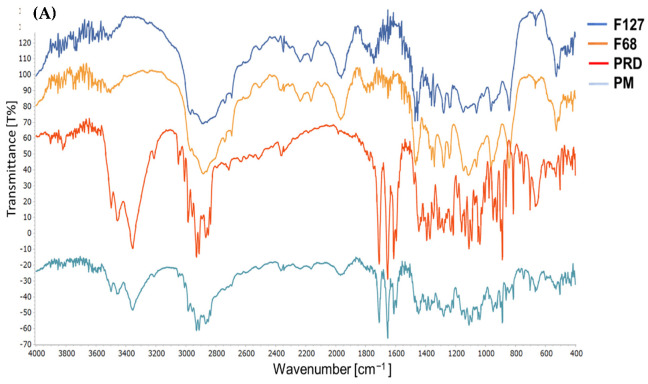

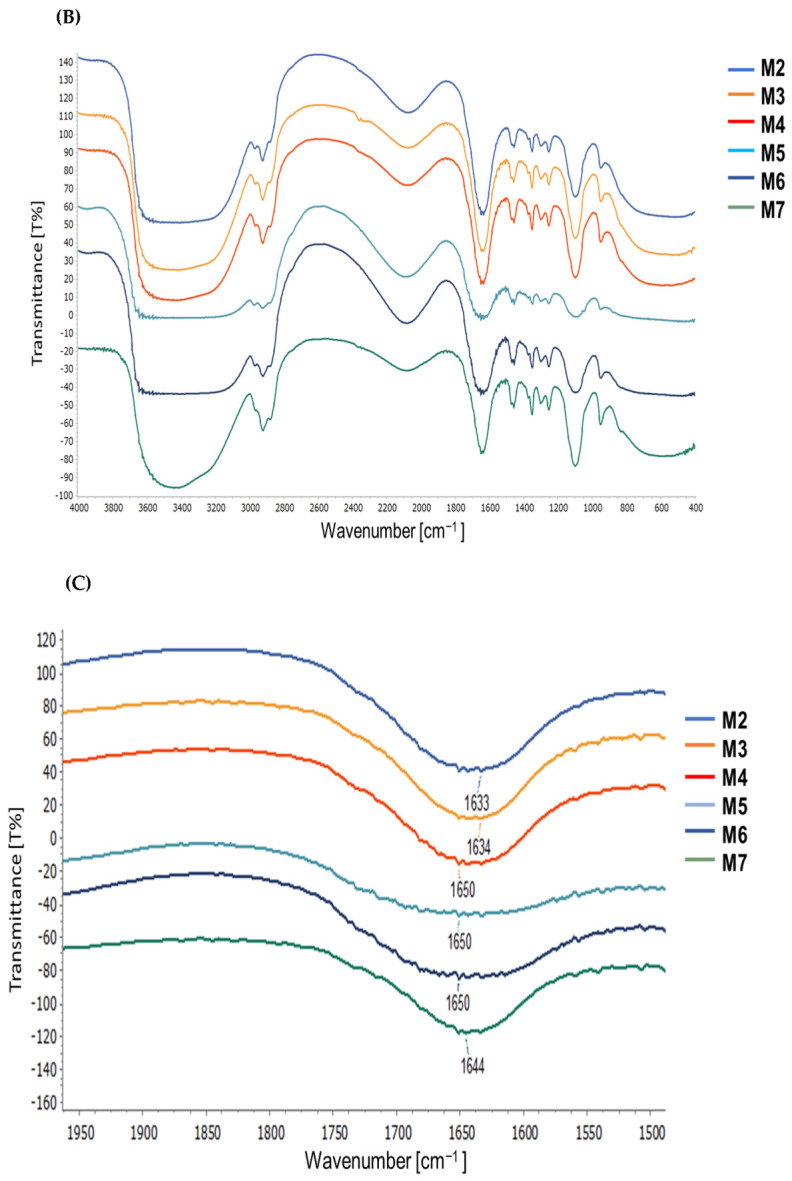
FTIR spectra of (**A**) F127: Pluronic^®^ F127, F68: Kolliphor^®^ P188, PRD: prednisolone, and PM: physical mixture, (**B**) M 2-7 microgels with shifted carbonyl stretching peak, and (**C**) shifted carbonyl stretching peaks in the M 2-7 microgels.

FTIR analysis was carried out to recognize the characteristic peaks of materials that would appear based on their chemical structure. Shifting, appearance, or disappearance of peaks indicate a chemical interaction between the materials used in the preparation. Figure 3A shows the FTIR spectra of PRD, F127, F68, and PM. For PRD, the three peaks at 3357, 3454, and 3496 cm−^1^ corresponded to the OH stretching peaks of PRD. The two peaks at 1710 and 1654 cm−^1^ corresponded to the C=O stretching of the two carbonyl peaks of PRD, following Palanisamy et al. [61]. The FTIR spectrum of F127 observed a sharp peak at 1344 cm−^1^, which corresponded to the O-H bending, and a C-H vibration peak observed at 2891 cm−^1^. Additionally, peaks at 1014, 1062, and 1130 cm−^1^ were assigned for C-O-C stretching vibrations. These observations are consistent with [62,63].

The FTIR spectrum of F68 observed a stretching vibration peak at 3504 cm−^1^, which corresponded to the OH group. Additionally, peaks in the 2890–2800 cm−^1^ region corresponded to the stretching vibration of C-H. For F127 and F68, a strong peak was visible at 1750 cm−^1^, corresponding to the C=O stretching and corroborating with previous studies [14,64].

In the FTIR spectrum of PM, the functional groups of PRD were dominantly exposed, although their intensity was reduced. Three peaks were visible at 3357, 3454, and 3496 cm−^1^, which corresponded to the OH stretching of PRD. Furthermore, two sharp peaks were observed at 1710 and 1654 cm−^1^, which might be attributed to the C=O stretching of PRD. The C=O stretching peaks of F127 and F68 were not visible, likely due to being masked by the sharp peaks of PRD. However, the absence of any chemical shifts in the peaks of PRD indicates the absence of any chemical interactions between PRD and either F127 or F68.

For PRD microgels (Figure 3B), the IR bands were broadened, shifted, or merged into broader peaks due to the presence of water. For F127, the C–O–C stretching vibration peaks, which appeared at 1015, 1063, and 1130 cm−^1^, were merged into a single broad band and shifted to lower frequencies of 900–1000 cm−^1^, indicating the formation of hydrogen bonds between oxygen atoms in the ether backbone of F127 and water molecules. The peaks observed at 2800–2890 cm−^1^, corresponding to the C-H stretching vibration for F127 and F68, were weakened and broadened to a lower extent [14]. The characteristic peaks for pure PRD, F127, and F68 were more visible in PM than those found in the microgels spectra, where the water was determined by a noticeable broadened peak in the HOH bending of 1500–1800 cm−^1^. Similar observations were discussed by Branca et al. [62].

Furthermore, a shift was observed in the carbonyl stretching peaks in M 2-7 microgels (Figure 3C). In microgels M2 and M3, the C=O stretching peak was seen at 1633 and 1634 cm−^1^, respectively. However, the C=O stretching peaks were shifted to 1650 cm−^1^ in M 4-6 microgels and to 1644 cm−^1^ in M7 microgel. The shift in the C=O stretching peaks indicated the formation of hydrogen bonding [65]. Furthermore, the shifting in the C=O stretching peaks and C–O–C stretching vibrations in the FTIR spectra of microgels indicated intermolecular interaction via hydrogen bonds and, hence, the formation of physically crosslinked microgels [66]. Additionally, as was previously discussed, the viscosity and viscoelastic properties of microgels were dramatically increased as the concentration of F68 was above 5%. This, coupled with the chemical shift observed in the FTIR data, strongly suggests that the mechanism of micelle formation is attributed to the formation of the water–poloxamer hydrogen bonds [65]. Table 6 summarizes the changes observed in the FTIR spectra of M 2-7 microgels.

#### 3.4.2. Differential Scanning Calorimetry (DSC)

**Figure 4:**
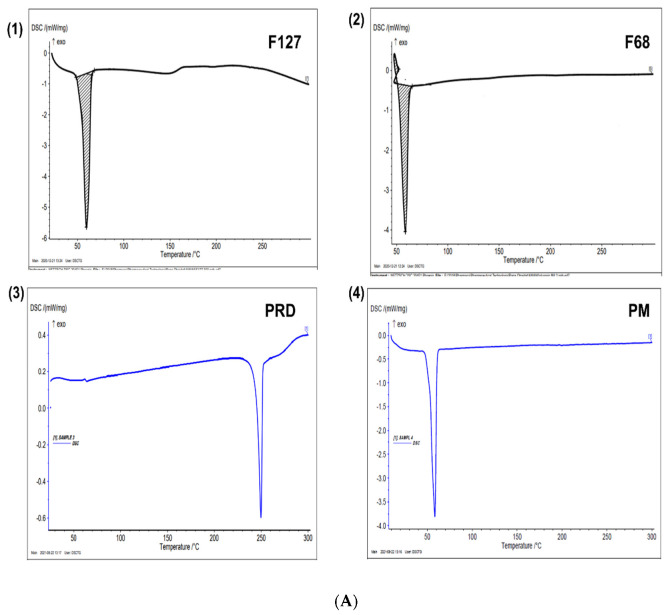

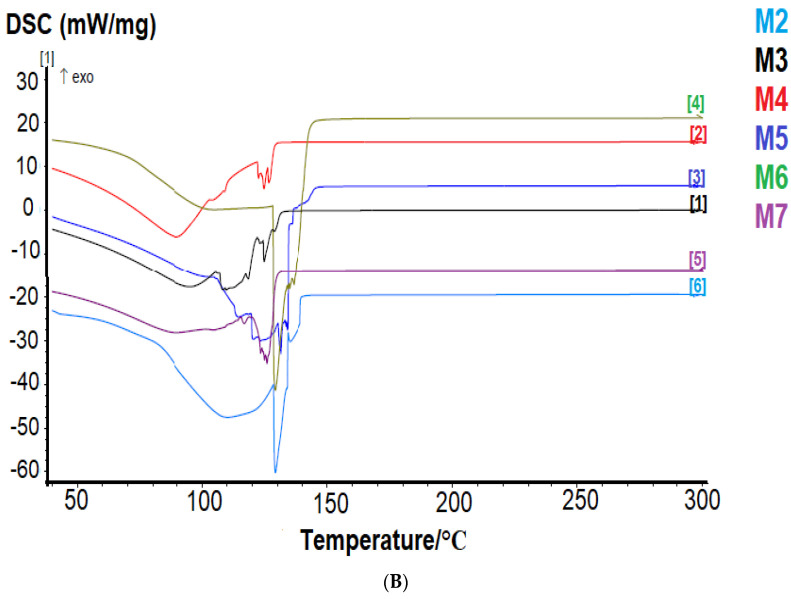
Differential scanning calorimetry (DSC) thermograms of (**A**) (**1**) F127: Pluronic^®^ F127, (**2**) F68: Kolliphor^®^ P188, (**3**) PRD: prednisolone, and (**4**) PM: physical mixture; and (**B**) M 2−7 microgels.

The DSC analysis was conducted for F127, F68, PRD powder, and PM (Figure 4A). The thermal DSC curves of F127 and F68 showed endothermic peaks at 59.7 and 58.5 °C, respectively, assigned to their melting points. Similar observations have been reported by Cavallari et al. [67]. The DSC curve of PRD showed a sharp endothermic peak at 250 °C, indicating the melting point of polymorph form III as reported by Veiga et al. [68]. The PM showed a single endothermic peak between 50 and 60 °C, corresponding to the melting points of F127 and F68. Additionally, the peak of PRD disappeared entirely, owing to the dispersion of the PRD into the molten polymers, as was previously reported by Obaidat et al. [69].

The DSC thermograms of M 2-7 microgels exhibited a broad peak at temperatures <100 °C, which is related to moisture due to the evaporation of the water phase in microgels (Figure 4B). Moreover, all thermal curves of microgels exhibited new peaks at 120–130 °C, which may be related to the polymeric interaction and the formation of MPM. Similar observations were reported by Sayed et al. [70], where an endothermic peak related to micellization appeared at 100 °C. The sharpness of this newly endothermic peak was increased with increasing F68 concentration, which further indicates that the micellization process of F68 was concentration-dependent. Splitting of the peaks appeared for all microgels except for the M6 microgel, composed of 7.5% F68, where a single peak appeared, indicating that this is the optimum concentration for the prepared MPM.

The results here can be correlated with T_sol-gel_ of microgels_,_ where the highest value was also observed for 7.5% F68 concentration. This observation requires further study to correlate the concentration of surfactants to the formation of MPM using thermal analysis. Furthermore, the absence of the PRD characteristic peak in the DSC thermogram of microgels confirms the complete entrapment of PRD within the molten polymeric micelles [71].

### 3.5. Rheological Studies

Rheological studies were assessed to study the viscosity and viscoelastic properties of M 2-7 microgels. Since these microgels are designed to be inserted into the conjunctival sac under the lower eyelid, the retention of microgels (ocular contact time) at the application site highly depends on their rheological properties [72]. Therefore, the relationship between the retention time and the rheological properties of microgels should be assessed since these systems will be transformed into gel state after exposure to the physiological temperature of the eye. The gel state would delay the clearance of microgels at the pre-ocular surface and prolong their retention time, improving drug bioavailability [73].

#### 3.5.1. Viscosity Curves

Typically, eye blinking produces a high shearing force on the applied formulations. The shear rate is about 0.03 s^−1^ during ocular interblinking periods and about 4250–28500 s^−1^ through blinking [74]. Therefore, the ideal ocular in situ systems should reveal high viscosity under low shear rates and low viscosity under high shear rates, representing a shear-thinning (pseudoplastic) behavior [74]. This is because if the viscosity increases with increasing shear rate (blinking), this will result in more eye irritation. Contrarily, if the viscosity is too low at a high shear rate, this will lead to increased drainage [75]. Therefore, to decrease drainage during blinking, ocular formulations should reveal a shear-thinning behavior with high viscosity at low shear rates [75].

**Figure 5:**
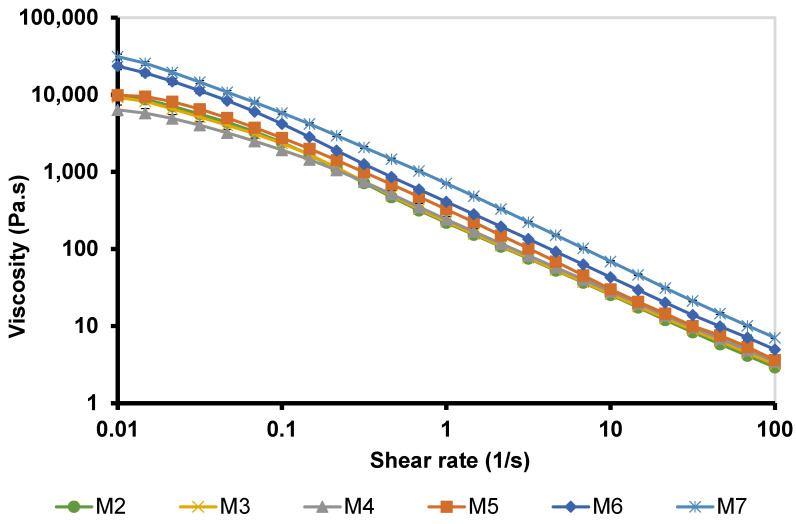
Viscosity curves of M 2-7 microgels at 35 °C and shear rates of 0.01–100 1/s.

The viscosity measurement of M 2-7 microgels was performed at 35 °C, within the ocular physiological temperature range of 32.8–35.4 °C [56]. The viscosity curves of microgels (viscosity vs. shear rate) exhibited a shear-thinning behavior, where the viscosity was high at low shear rates and then decreased with increasing the applied shear rates (Figure 5).

M2 (12% F127) and M3 (12% F127/1% F68) microgels exhibited similar viscosities, where the viscosity curves were superimposed. Hence, adding 1% F68 did not influence the viscosity of microgels. The addition of 2.5% F68 slightly reduced the viscosity of M4 microgel compared to those of M 2-3 microgels. Additionally, increasing the concentration of F68 increased the viscosity of microgels in the order: M5 (5% F68) < M6 (7.5% F68) < M7 (10% F68). These results agree with Pawar et al. [74], who reported that increasing the concentration of F68 in the in situ systems can lead to micellar entanglement and change the PEO/PPO ratio, hence forming a more viscous gel.

#### 3.5.2. Mathematical Modeling

The viscosity data of M 2-7 microgels, fitted into the Carreau–Yasuda and Casson models, displayed a good correlation coefficient (R^2^) of 0.9981–0.9997 and 0.9662–0.9983, respectively. The calculated rheological parameters obtained from the models are summarized in Table 7. The rheological parameter yield stress $\left(\tau ο\right)$ can be used to describe the flow behavior of the non-Newtonian systems, where below$\;\left(\tau ο\right)$, the material behaves as a solid, and above $\left(\tau ο\right)$, the material behaves as a liquid [76]. All PRD microgels exhibited $\left(\tau ο\right)$ values in the range of 231.49- 657.36 Pa, where microgels start to flow at these values, suggesting a shear-thinning behavior [36]. Binsi PK et al. [77] reported that$\;\left(\tau ο\right)$ is dependent on both concentration and temperature, where the $\left(\tau ο\right)\;$values of M 3-7 microgels, which are composed of F127/F68, were higher than that of M2 microgel, which is composed of F127 only. Furthermore, the effect of F68 on $\left(\tau ο\right)$ was concentration-dependent, where increasing the F68 concentration resulted in an increase in$\;\left(\tau ο\right),$ suggesting a more rigid microgel structure. These results are consistent with Contreras et al. [78] who found that increasing Carbopol concentration significantly increased the crosslinking and rigidity of Carbopol networks, resulting in high yield stresses.

#### 3.5.3. Strain–Sweep

The LVR is the region of low oscillatory strains, where the elastic (G′) and viscous (G″) moduli of microgels remained constant with increasing strains. Thus, microgels remained intact and G′ and G″ are mainly related to the molecular structure of microgels [79]. The LVRs of M 2-7 microgels are illustrated in Table 8. Specific critical strains (γ_c_), within the LVR, of 0.04 for M 2-3 and 0.10 for M 4-7 microgels were selected for the subsequent frequency–sweep studies.

#### 3.5.4. Frequency–Sweep

**Figure 6:**
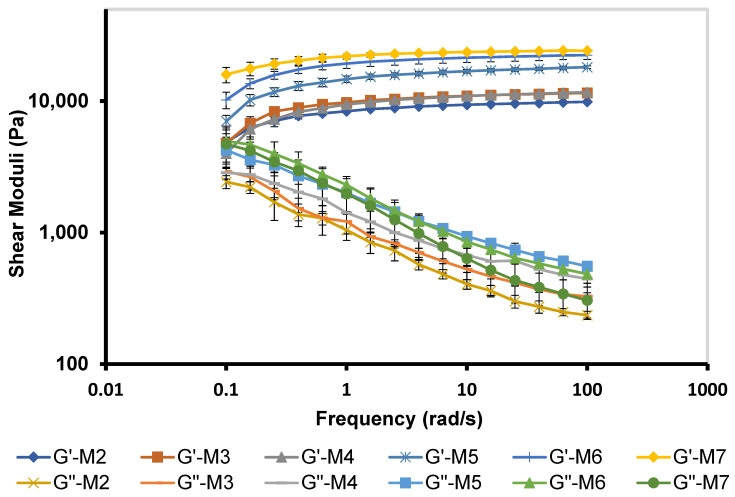
Frequency-dependent elastic (G′) and viscous (G″) moduli of M 2-7 microgels over a frequency range of 0.1–100 rad/s.

The viscoelastic properties of M 2-7 microgels were characterized by G′ and G″ and assessed by subjecting the microgels to various frequencies (1–100 rad/s) (Figure 6). Microgels exhibited viscoelastic networks with G′ dominated G″ over the frequency range, suggesting higher ocular retention of microgels and prolonged drug release profiles [75]. In addition, M 2-7 microgels showed a frequency-dependent elastic behavior, where G′ increased with an increase in frequency [80]. Furthermore, G′ and G″ of microgels increased with an increase in the F68 concentration in the order: M7 (10% F68) > M6 (7.5% F68) > M5 (5% F68), which corroborated the viscosity data. Edsman et al. [75] found that the ocular Carbomer gels with high G′ are less susceptible to deform. As a result, they exhibited increased gel retention that remained under the eyelid without any flow toward the cornea. This, in turn, protects the eye from blurred vision and decreases blinking.

#### 3.5.5. Temperature–Sweep

**Figure 7:**
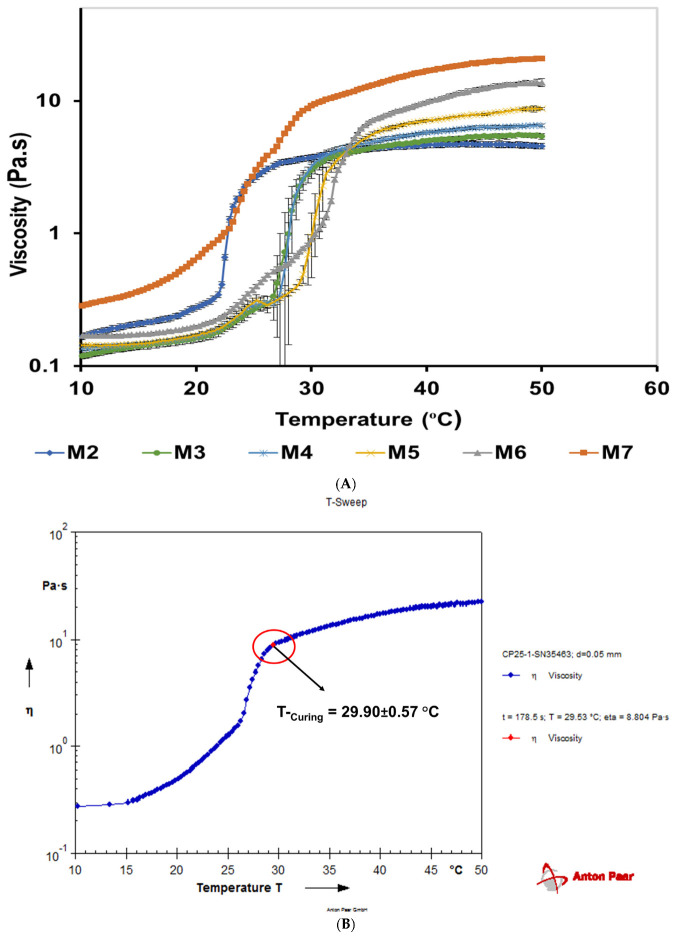

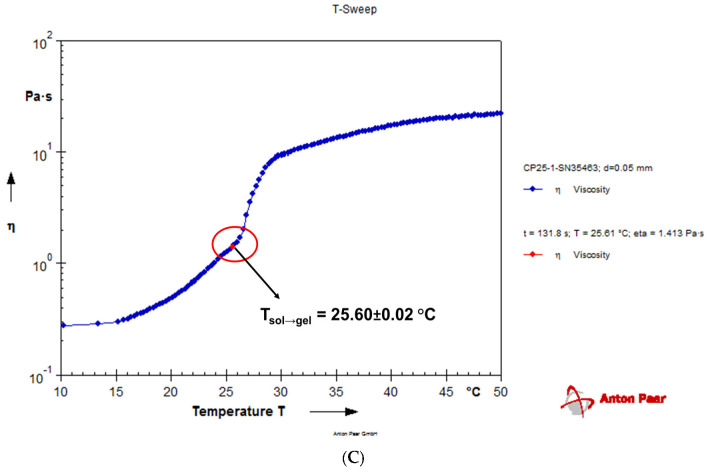
(**A**) The temperature–sweep tests illustrating the phase transition profiles of M 2−7 microgels determined using controlled-stress rheometer (CRS), (**B**) determination of the sol transition temperature (T_sol→gel_) of M7 microgel from its phase transition profile as indicated by an arrow, and (**C**) determination of the curing temperature (T-_Curing_) of M7 microgel from its phase transition profile as indicated by an arrow.

The thermoresponsive in situ formulations for ocular delivery need to gel when administered onto the eye at a temperature range of 30–35 °C [57]. CRS was utilized to determine the T_sol→gel_ and curing temperature (T-_Curing_) of M 2-7 microgels from the temperature–sweep tests (viscosity vs. temperature) during the phase transition (Figure 7) [81]. Initially, the viscosity was low owing to the sol state of the microgels; then, it increased drastically with increasing temperature due to gel formation. At the gel state, the phase transition from sol to gel occurred, and the viscosity became independent of a further increase in temperature (Figure 7A).

For M 2-7 microgels, the T_sol→gel_ was determined when the phase transition from sol-to-gel started [10], whereas T-_Curing_ was determined when the gel point was identified and the microgels were solidified [82]. Figure 7B,C illustrate the T_sol→gel_ (25.6 ± 0.0 °C) and T-_Curing_ (29.9 ± 0.6 °C) of the M7 microgel, respectively, determined from the temperature–sweep test during phase transition. Table 9 shows the T_sol→gel_ measured by the tube inversion method and CSR, and the T-_Curing_ determined using the CSR for M 2-7 microgels_._ Data showed that the T_sol→gel_ of microgels, assessed by the tube inversion method, were close to T_Curing._ This is because, in the tube inversion method, the T_sol→gel_ was recorded when the microgel was fully converted to gel state, thus aligning with the gel points of microgels (T_Curing_).

### 3.6. Mucoadhesion Studies

Mucoadhesive characteristics of M 2-7 microgels were evaluated by determining the rheological synergism between microgels and mucins [24]. Understanding the mucoadhesive interactions between the formulations and ocular mucosa is a crucial step during the development of ocular delivery systems to enable a longer contact time between the formulation and the corneal surface, thus increasing drug bioavailability [83]. This is because the typical mucosal layer exhibits a gel layer composed of randomly entangled high-molecular-weight glycoproteins (mucins) at a concentration range of 2–5% *w/w* with an average thickness of 3–5 µm on the cornea [84,85]. Thus, the rheological synergism between microgels and mucins evaluated the ability of the thermoresponsive polymers (F127 and F68), used in the preparation of microgels, to adhere to the ocular mucosa by measuring the increase in viscosity or viscoelasticity after mixing with mucins.

In this study, the rheological synergism of mucins was initially studied at different mucin concentrations (2, 2.5, 3, and 5%), close to those reported in the mucosal layer of the eye [84,85]. These primary studies would determine the effect of mucin concentrations on the dynamic moduli G′ and G″ and choose the best mucins concentrations to carry out the mucoadhesive studies.

**Figure 8:**
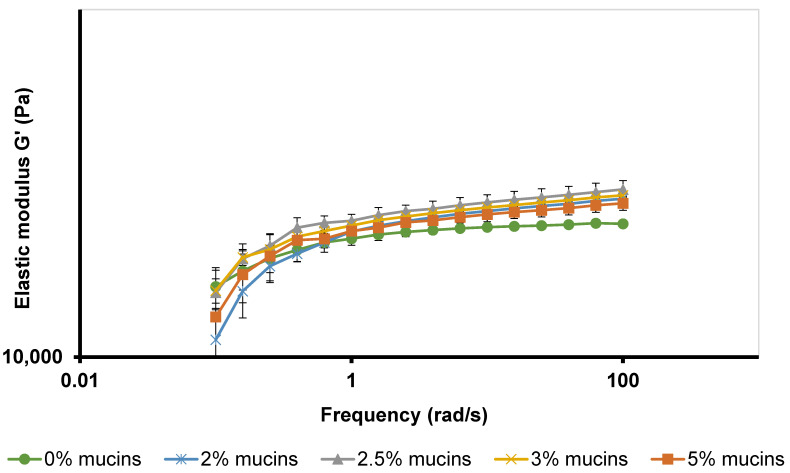
Frequency-dependent elastic modulus (G′) of M7 microgel after mixing with various mucins concentrations at 35 °C.

Owing to its highest viscoelastic properties (G′ and G″), M7 microgel was selected to evaluate the mucoadhesive interactions between microgels and different mucin concentrations. Figure 8 shows the frequency-dependent G′ of M7 microgel after mixing with 2, 2.5, 3, and 5% mucin dispersions. All mucins/M7 microgel mixtures showed higher G′ values than those of M7 microgel. Moreover, mixing M7 microgel with 2.5% mucins dispersion gave the highest G′ over frequency of 0.1–100 rad/s. Thus, a mucin concentration of 2.5% was selected for evaluating the rheological synergism and calculating the bioadhesive interaction parameter (ΔG′) for M 2-7 microgels.

Table 10 illustrates the calculated mucoadhesive interaction parameter (ΔG′) by subtracting the G′ values of mucins/M 2-7 microgels mixtures from their corresponding microgels at 1, 10, and 100 rad/s. The rheological synergism for mucins/M2 microgel mixture was frequency-dependent, where positive and negative interactions were found based on the frequency value. For instance, a negative interaction (ΔG′ = −1517.5 Pa) was found at low frequency (1 rad/s), a weak positive interaction (ΔG′ = +143.3 Pa) was found at 10 rad/s, and a strong positive interaction (ΔG′ = +1820 Pa) was reported at 100 rad/s. In addition, a strong positive interaction was observed in mucins/M 3-7 microgels mixtures, particularly for M 6-7 microgels, at all frequencies (1, 10, and 100 rad/s), indicating that 2.5% mucin dispersions interact with microgels with high mucoadhesiveness.

### 3.7. Eye Irritation

**Figure 9:**
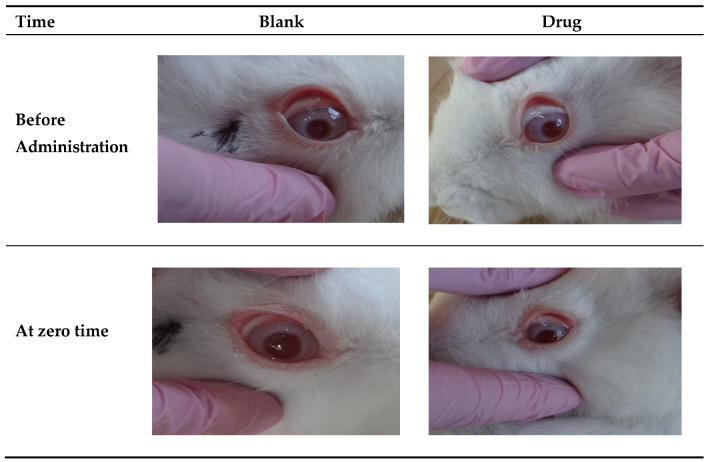

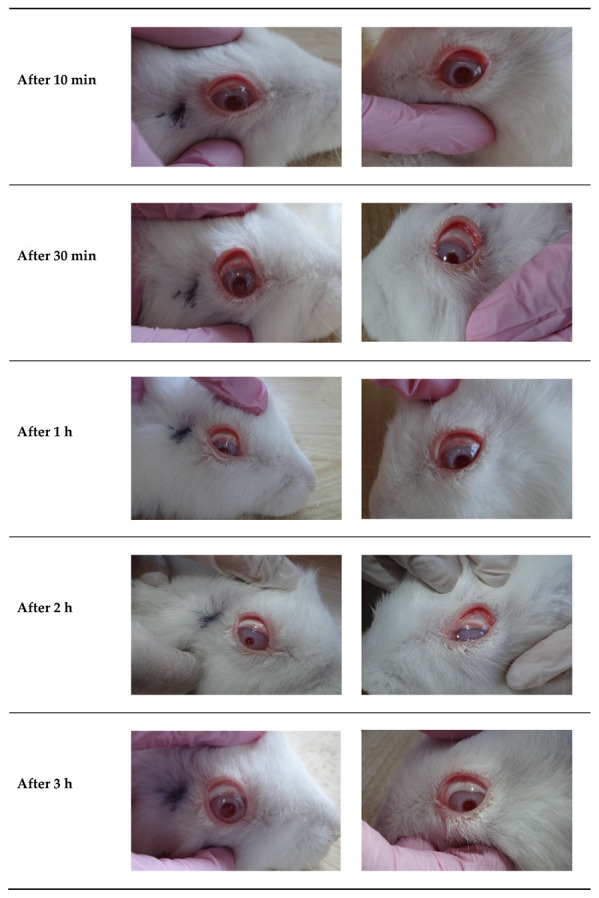

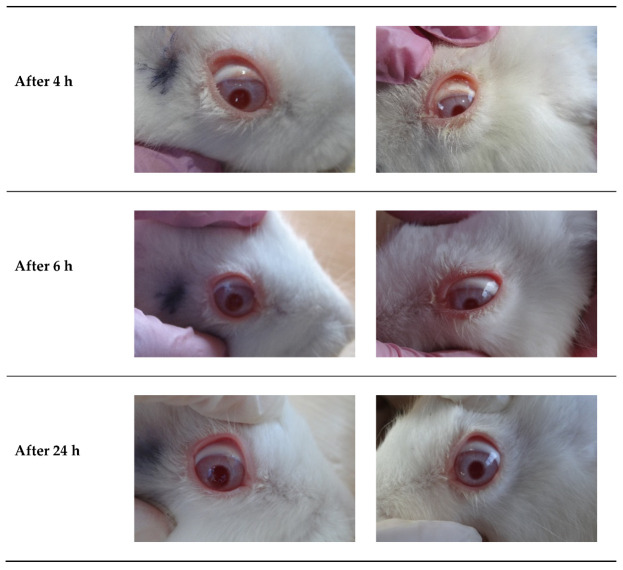
Results of irritation test at different time intervals after the administration of blank and M7 microgel.

The selected M7 microgel was applied in a rabbit’s right eye while the blank microgel (without drug) was administered in the left eye as a control. The M7 microgel with the highest F68 concentration (10%) was selected based on its highest viscoelastic properties. Edsman et al. [75] reported that gels with higher Carbopol concentration showed no irritation effect. This was attributed to the drastic increase in the gel’s elasticity and strength. Thus, the gel would stay in place without flowing onto the sensitive cornea. Therefore, two positive effects can be achieved in response to the increase in gel elasticity: longer contact time and elimination of irritation [75]. As shown in Figure 9, the blank microgel revealed a slight eye redness with no conjunctival chemosis or discharge at the first time of instillation. Based on the modified Draize scores, only grades 0 and occasionally 1 were recorded. After 2 h of administration, the conjunctival redness completely disappeared. Although the rabbit eye is more susceptible to irritation compared to human eyes [86], it was found that the microgel was suitable for the ocular application, causing no irritation and is well-tolerated. Moreover, it was observed that the eye redness was less in the presence of the drug compared to blank microgel, suggesting the role of PRD as an anti-inflammatory agent in reducing the eye irritation caused by formulation components [87].

### 3.8. Drug Assay

The PRD content in M 2-7 microgels was 100.4–105.2% (Table 11), indicating that the drug was uniformly distributed throughout the microgels.

### 3.9. In Vitro Release

In the in vitro release studies, sink condition was maintained in the release medium even if the total amount of PRD (2.5 mg) was released from 1 g of microgels placed in the donor phase. This is because the solubility of PRD in the release medium was tenfold more than the amount of PRD in 1 g of microgel.

**Figure 10:**
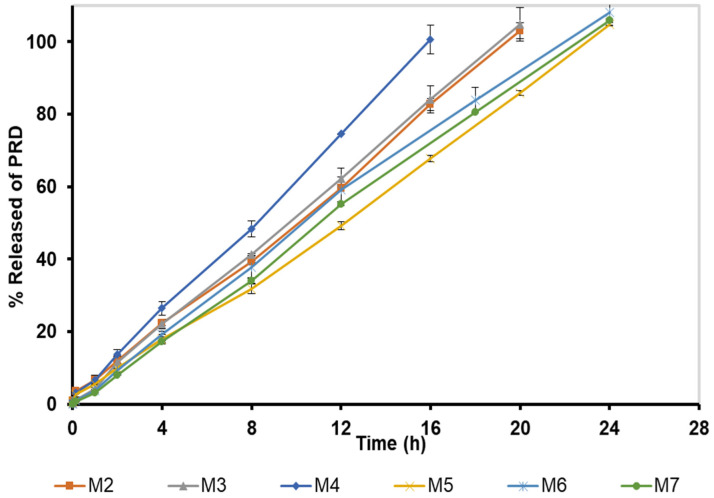
In vitro release profiles of M 2-7 microgels through 24 h at 35 °C.

M 2-7 microgels showed sustained drug release profiles for 16–24 h (Figure 10). M2 and M3 microgels, which contain 12% F127 and 12% F127/1% F68, respectively, nearly exhibited the same release profiles, where the drug release rate increased with time, reaching >100% after 20 h. The M4 microgel, which contains 12% F127/2.5% F68, showed a faster release rate with a complete drug release after 16 h. This might be attributed to its low viscosity and viscoelastic properties (Figure 5 and Figure 6). In comparison, the in vitro release of PRD from M 5-7 microgels was completed after 24 h of 104.7 ± 0.43, 107.9 ± 3.4, and 105.9 ± 0.45%, respectively. The release results of M 5-7 microgels agree with their rheological properties (viscosity and viscoelasticity), where viscous and high-viscoelastic microgels exhibited slower-release profiles. Rajalakshmi et al. [88] reported that the increase in gel viscosity could explain the slow drug release profile of Poloxamer-based in situ gels for ocular delivery.

### 3.10. Mechanism of Drug Release

The mechanism of drug release from M 2-7 microgels was determined by fitting the release data into zero-order, first-order, Higuchi, and Korsmeyer–Peppas models. The logarithm of the % release of PRD and logarithm of time were fitted with the linear regression analysis using Excel [89]. Based on the coefficient of determination (R^2^), the Korsmeyer–Peppas model showed a good fitting for the drug release data of M 2-7 microgels with R^2^ in the range of 0.9946–0.9997 (Table 12). The values of “n” indicate the release mechanism for the drug, where n ≤ 0.5 indicates a Fickian diffusion, *n* = 1 indicates a zero order, and n between 0.5 and 1 indicates a non-Fickian diffusion, where the release is controlled by diffusion and erosion of the polymeric chain [90]. The rate of PRD from M2 microgel, composed of F127 only, was controlled by diffusion and erosion since n ≈ 0.88. The PRD release mechanism from M 3-7 microgels, composed of F127 and F68, followed a zero-order release since n ≈ 1. Thus, the rate of drug release from M 3-7 microgels was constant despite the concentration of PRD.

### 3.11. Stability Studies of PRD Microgels

#### 3.11.1. Thermal Stability

The appearance and drug content of PRD microgels were assessed initially and after three months of storage at room temperature, 30 °C/65% RH, and 40 °C/75% RH. The microgels remained stable without precipitation or turbidity except for M 2-3 microgels, where slight turbidity was observed after three months of storage at 40 °C/75% RH. Additionally, the PRD content in M 2-7 microgels was analyzed by HPLC. The results showed no significant difference in the PRD content of M 2-7 microgels stored at room temperature and 30 °C/65% RH for three months compared with those initially assessed at zero time, indicating that the drug was stable during storage (Table 11). Moreover, no change in the eluted time and no appearance of new peaks were observed when the HPLC analysis was carried out for the stored microgels, which further emphasized that PRD microgels were chemically stable. However, the drug content of M 2-7 microgels decreased from 80.8 ± 4.0 to 92.3 ± 1.9% after three months of storage at 40 °C/75% RH. This reduction might be attributed to the higher temperature and RH.

#### 3.11.2. Size and Zeta Potential

In general, a change in size during storage is likely due to the coalescence of droplets [91]. The maximum MPM size was recorded for the M6 microgel at 58.3 ± 1.2 nm. Additionally, no change was found in ZP after three months of storage at room temperature (Table 5). Therefore, the results showed that M 2-7 microgels were stable for three months in size and ZP, where no significant change in size or ZP was observed due to the absence of coalescence or aggregation.

#### 3.11.3. Rheological Stability

The rheological stability of M 2-7 microgels was assessed for T_sol→gel_, T_(gel),_ viscosity, and viscoelastic properties after six months of storage at room temperature.

##### T_sol→gel_ and T_(gel)_

The T_sol→gel_ and T_(gel)_ of M 2-7 microgels after six months of storage at room temperature are illustrated in Table 9. Data showed no difference between the initial data of T_sol→gel_ and T_(gel)_ of M 2-7 microgels and those reported for stored microgels.

##### Viscosity and Viscoelastic Properties

**Figure 11:**
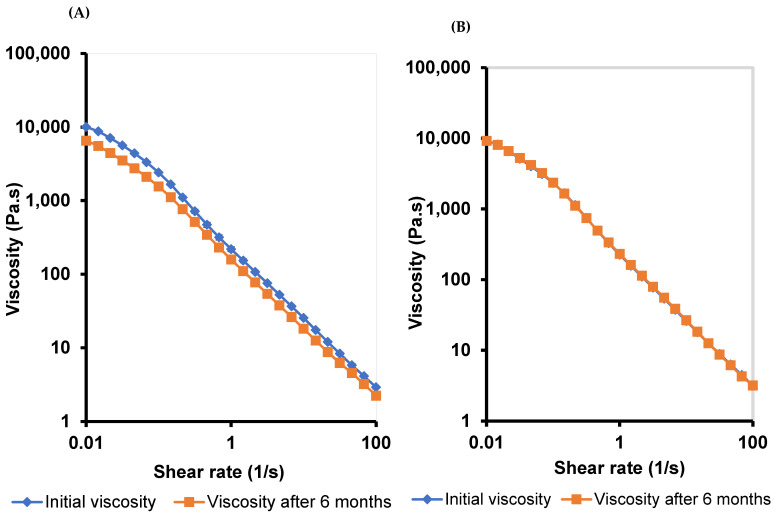

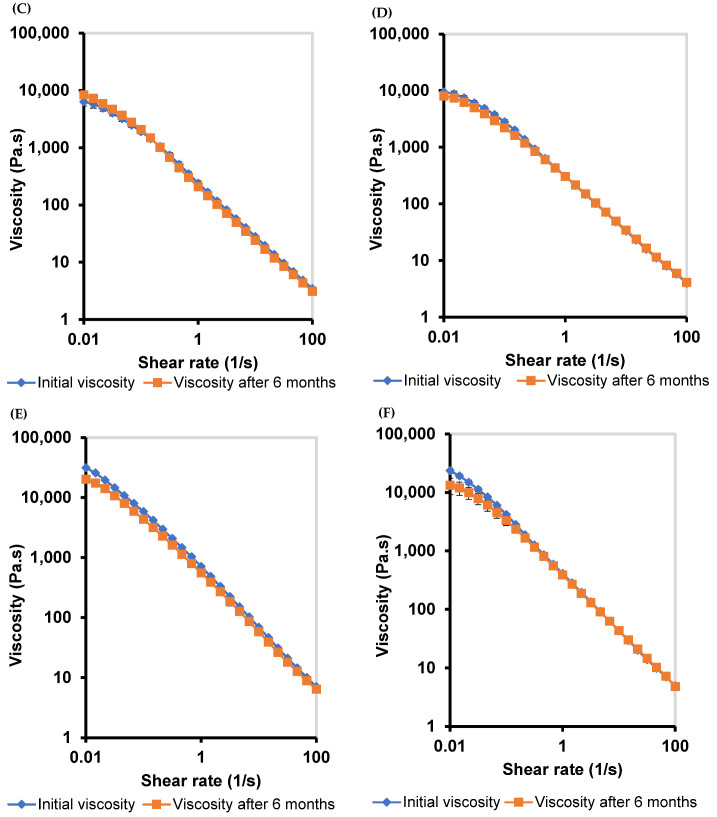
Viscosity curves of: (**A**) M2, (**B**) M3, (**C**) M4, (**D**) M5, (**E**) M6, and (**F**) M7 microgels after six months of storage at room temperature.

To further investigate the stability of microgels, viscosity curves were evaluated after six months of storage at room temperature. The M2 microgel showed a slight reduction in viscosity after six months (Figure 11A). This might be attributed to its low viscosity. The M 3-6 microgels showed no difference in viscosity, indicating that these microgels maintained their integrity (Figure 11B–E). In addition, a slight decrease in viscosity was also found in the M7 microgel (Figure 11F). Furthermore, the viscosity data of microgels subjected to the stability study were fitted well to Carreau–Yasuda and Casson models with R^2^ of 0.9979–0.9997 and 0.9145–0.9965, respectively. The estimated values of $\left(\eta ο\right)$, $\left(\eta \infty \right)$, $\left(\tau ο\;\right),\;$and R^2^ after six months of storage are summarized in Table 7.

**Figure 12:**
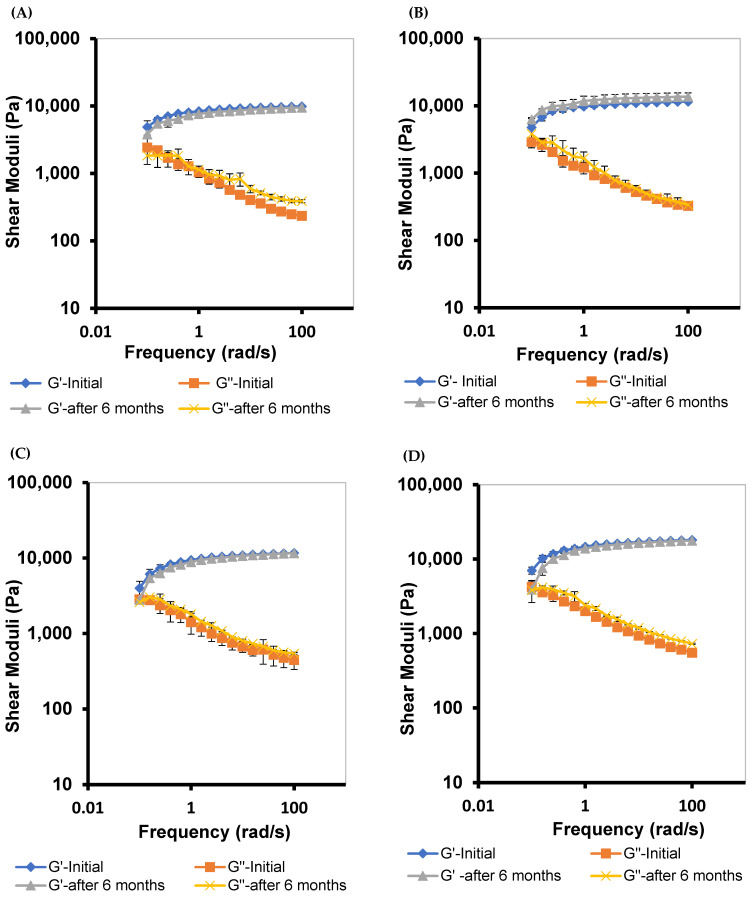

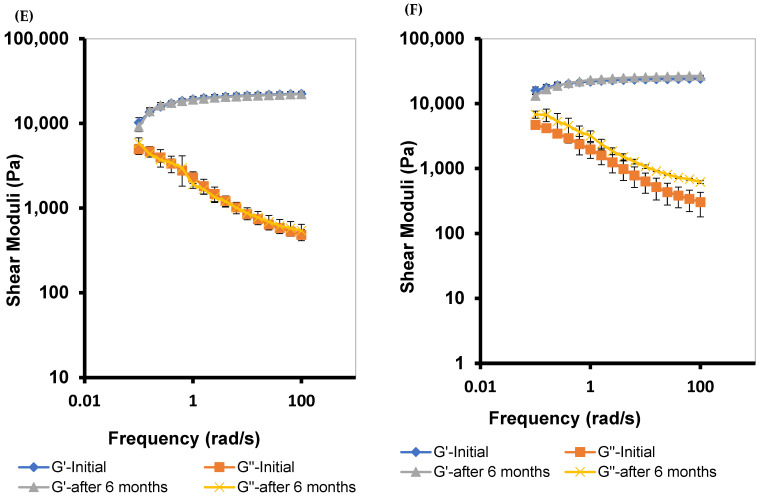
Frequency-dependent elastic (G′) and viscous (G″) moduli of: (**A**) M2, (**B**) M3, (**C**) M4, (**D**) M5, (**E**) M6, and (**F**) M7 microgels after six months of storage at room temperature.

Additionally, the M 2-7 microgels showed no reduction in the viscoelastic properties after six months of storage at room temperature, indicating that the microgels maintained their integrity (Figure 12). This is because F68 is an essential component in the polymeric hydrogel and the PRD-MEs, thus enhancing the stability of PRD microgels.

## 4. Conclusions

PRD-MEs, proposed as lipid nanosystems to enhance the solubility of PRD, were combined with the thermosensitive in situ polymeric F127 and F68 hydrogels to achieve a sustained local drug delivery to the eye. The potential of PRD loaded into in situ microgels as drug carriers for ocular delivery was evaluated. The clarity, pH, surface tension, size, and zeta potential of MPM, T_sol-gel,_ T_(gel),_ and drug content of optimized PRD microgels were satisfactory for their intended use. A combination of F127 and F68 formed transparent PRD microgels that converted from sol state to gel state at a temperature close to the eye’s surface. This work highlighted the role of the combined system of PRD-ME and in situ hydrogels in enhancing the mechanical and mucoadhesive properties, prolonging ocular retention time, and increasing the stability of microgels. Additionally, PRD microgels were nonirritant to mucosal tissues, suggesting the suitability of these systems for local ocular delivery. Moreover, PRD microgels could control the drug release for up to 24 h.

## Figures and Tables

**Table 1:** Composition and visual testing of the pseudoternary systems.

System	*S_mix_ (Ratio, *w*/*w*%)	OA (*w*/*w*%)	Water (*w*/*w*%)	Pass/Fail the Visual Test
S1	TW-20: Ethanol (1:1, 55)	10	35	Pass
S2	TW-20: Ethanol (1:2, 55)	10	35	Pass
S3	TW-20: Ethanol(2:1, 55)	10	35	Pass
S4	TW-20: PG (1:1, 55)	10	35	Pass
S5	TW-20: PG (1:2, 55)	10	35	Pass
S6	TW-20: PG (2:1, 55)	10	35	Pass
S7	LB: Ethanol (1:1, 55)	10	35	Fail
S8	LB: Ethanol (1:2, 55)	10	35	Fail
S9	LB: Ethanol (2:1, 55)	10	35	Fail
S10	LB: PG (1:1, 55)	10	35	Fail
S11	LB: PG (1:2, 55)	10	35	Fail
S12	LB: PG (2:1, 55)	10	35	Fail

*S_mix_: a combination of surfactant and cosurfactant; TW-20: Tween^®^ 20; PG: propylene glycol; LB: Labrasol; OA: oleic acid.

**Table 2:** Composition of PRD-MEs.

*PRD-ME	PRD (%)	S_mix_ (Ratio, *w*/*w*%)	OA (*w*/*w*%)	Water (*w*/*w*%)	F68 (40%) (*w*/*w*%)	Observations
1	1	TW-20: Ethanol (1:1, 55)	10	35	-	Precipitation of PRD
2	1	TW-20: Ethanol (1:2, 55)	10	35	-	Precipitation of PRD
3	1	TW-20: Ethanol (2:1, 55)	10	35	-	Precipitation of PRD
4	1	TW-20: PG (1:1, 55)	10	35	-	Precipitation of PRD
5	1	TW-20: PG (1:2, 55)	10	35	-	Precipitation of PRD
6	1	TW-20: PG (2:1, 55)	10	35	-	Precipitation of PRD
7	0.25	TW-20: Ethanol (1:2, 55)	10	-	35	Clear
8	0.5	TW-20: Ethanol (1:2, 55)	10	-	35	Clear
9	0.75	TW-20: Ethanol (1:2, 55)	10	-	35	Clear
10	1	TW-20: Ethanol (1:2, 55)	10	-	35	Precipitation of PRD

*PRD-MEs: prednisolone nanoemulsions; PRD: prednisolone; S_mix_: a combination of surfactant and cosurfactant; TW-20: Tween^®^ 20; PG: propylene glycol; LB: Labrasol; OA: oleic acid; Kolliphor^®^ P188: F68.

**Table 3:** Composition and _Tsol→gel_ of PRD microgels.

PRD Microgels	*PRD (%)	F127 (%)	F68 (%)	Clarity	pH Mean ± SD	Surface Tension (mN/m)	T_sol→gel_ (°C) Mean ± SD	T_(gel)_ (min) Mean ± SD
M1	0.25	10	-	Not clear	Not reported	Not reported	32.0 ± 0.5	-
M2	0.25	12	-	Clear (+)	5.45 ± 0.08	31.4 ± 0.1	26.0 ± 0.7	1.47 ± 0.03
M3	0.25	12	1	Clear (++)	5.58 ± 0.03	31.7 ± 0.8	30.5 ± 0.5	2.05 ± 0.03
M4	0.25	12	2.5	Clear (++)	5.61 ± 0.04	35.4 ± 1.2	31.2 ± 0.7	2.22 ± 0.03
M5	0.25	12	5	Clear (++)	5.76 ± 0.05	31.2 ± 0.1	33.0 ± 0.5	2.30 ± 0.08
M6	0.25	12	7.5	Clear (++)	5.43 ± 0.06	37.0 ± 1.9	35.0 ± 1.0	2.35 ± 0.12
M7	0.25	12	10	Clear (++)	5.71 ± 0.05	36.4 ± 2.0	30.0 ± 0.5	1.52 ± 0.35
M8	0.5	12	-	Not Clear (Precipitation of PRD)	Not reported	Not reported	Not reported	Not reported
M9	0.5	12	7.5	Not Clear (Precipitation of PRD)	Not reported	Not reported	Not reported	Not reported
M10	0.5	12	10	Not Clear (Precipitation of PRD)	Not reported	Not reported	Not reported	Not reported
M11	0.75	12	-	Not Clear (Precipitation of PRD)	Not reported	Not reported	Not reported	Not reported
M12	0.75	12	7.5	Not Clear (Precipitation of PRD)	Not reported	Not reported	Not reported	Not reported
M13	0.75	12	10	Not Clear (Precipitation of PRD)	Not reported	Not reported	Not reported	Not reported

*PRD: prednisolone; Kolliphor^®^ P188: F68; Pluronic^®^ F127: F127; T_sol→gel_: sol–gel transition temperature; T_(gel):_ gelation time; Clear (+): sufficiently clear PRD microgels; Clear (++): highly clear PRD microgels.

**Table 4:** Mean droplet size (MDS), polydispersity index (PDI), and zeta potential (ZP) of prednisolone microemulsion 7 (PRD-ME 7) as a function of time. Data are presented as mean ± SD (*n* = 3).

Time	Initial	1 Week	2 Weeks	3 Weeks	One Month
MDS (nm)	16.4 ± 2.2	18.7 ± 2.8	20.6 ± 2.8	39.8 ± 4.7	67.9 ± 1.9
PDI	0.24 ± 0.01	0.35 ± 0.01	0.31 ± 0.01	0.30 ± 0.04	0.30 ± 0.02
ZP (mV)	−21.03 ± 1.24	−14.40 ± 0.07	−14.16 ± 2.21	−12.60 ± 1.50	−9.71 ± 0.60

**Table 5:** Size and zeta potential (ZP) of the mixed polymeric micelle (MPM) of PRD M 2-7 microgels after three months of storage at room temperature. Data are presented as mean ± SD (*n* = 3).

PRD Microgels	Initial		Three Months	
	Size (nm)	ZP (mV)	Size (nm)	ZP (mV)
M2	22.1 ± 0.9	−4.77 ± 0.47	53.9 ± 0.2	−4.59 ± 0.92
M3	21.8 ± 0.2	−8.15 ± 1.36	39.1 ± 2.8	−6.22 ± 0.73
M4	20.2 ± 0.1	−8.95 ± 1.30	50.5 ± 2.7	−4.22 ± 0.65
M5	20.7 ± 0.2	−6.23 ± 0.03	53.0 ± 2.7	−2.56 ± 0.38
M6	23.0 ± 0.9	−5.11 ± 0.99	58.3 ± 1.2	−4.00 ± 0.50
M7	21.3 ± 0.1	−2.13 ± 0.79	52.0 ± 0.8	−4.31 ± 0.34

**Table 6:** The changes observed in the FTIR spectra of M 2-7 microgels.

PRD Microgels	C-H Stretching Peak (cm−^1^)	C–O–C Stretching Vibration Peak (cm−^1^)	C=O Stretching Vibration Peak (cm−^1^)
M2	2994	995.5	1633
M3	2995	990.6	1634
M4	2996	990.6	1650
M5	3000	987.6	1650
M6	2997	990.0	1650
M7	2923	985.8	1644

**Table 7:** The rheological parameters (infinite shear rates $\left(\eta ο\right)$, infinite viscosity $\left(\eta \infty \right)$, yield stress $\left(\tau ο\right),\;$ and correlation coefficient (R^2^)) of the PRD microgels obtained using the mathematical models Carreau–Yasuda and Casson.

PRD Microgels	Carreau–Yasuda Model					Casson Model				
	Initial			After Six Months			Initial		After Six Months	
	ηο (mPa·s)	η∞ (mPa·s)	R^2^	ηο (mPa·s)	η∞ (mPa·s)	R^2^	το (Pa)	R^2^	το (Pa)	R^2^
M2	1.10 × 10^7^	255.61	0.9982	9.02 × 10^6^	578.29	0.9986	231.49	0.9662	165.22	0.9884
M3	1.07 × 10^7^	810.16	0.9981	1.08 × 10^7^	8750	0.9979	243.66	0.9983	220.23	0.9145
M4	8.00 × 10^6^	615,74	0.9992	1.04 × 10^7^	803.67	0.9993	258.76	0.9951	211.94	0.9667
M5	1.08 × 10^7^	606.8	0.9995	1.29 × 10^7^	186.15	0.9995	314.72	0.9224	334.85	0.9965
M6	3.08 × 10^7^	726.81	0.9997	1.59 × 10^7^	746.6	0.9997	389.56	0.9640	370.68	0.9900
M7	9.17 × 10^7^	0.1	0.9995	3.58 × 10^7^	684.08	0.9997	657.36	0.9012	481.29	0.9257

**Table 8:** Linear viscoelastic region (LVR) and critical strain (γ_c_) of PRD microgels at 35 °C.

PRD Microgels	LVR	γ_c_
M2	0.01–0.251	0.04
M3	0.0158–0.251	0.04
M4	0.0251–0.251	0.10
M5	0.0398–0.398	0.10
M6	0.01–0.398	0.10
M7	0.01–0.398	0.10

**Table 9:** Sol–gel transition temperature (T_sol→gel_) determined by the tube inversion and temperature–sweep methods, gelation time (T_(gel)_)**_,_** curing temperature (T_Curing_) determined by the temperature–sweep method, and T_sol→gel_ and T_(gel)_ of the PRD microgels after six months of storage at room temperature. Data are presented as mean ± SD (*n* = 3).

PRD Microgels	T_sol→gel_ (°C) by Tube Inversion Method		T_(gel)_ (min)		Temperature–Sweep Test	
	Initial	Six Months	Initial	Six Months	T_sol→gel_ (°C)	T-_Curing_ (°C)
M2	26.0 ± 0.7	25.0 ± 0.3	1.6 ± 0.1	1.5 ± 0.1	21.40 ± 0.17	26.79 ± 0.17
M3	30.5 ± 0.5	30.0 ± 0.7	1.5 ± 0.0	1.4 ± 0.1	25.73 ± 0.24	30.78 ± 0.40
M4	31.2 ± 0.7	30.5 ± 0.5	2.2 ± 0.1	2.0 ± 0.2	26.74 ± 0.15	30.62 ± 0.58
M5	33.0 ± 0.5	33.0 ± 0.5	2.4 ± 0.1	2.2 ± 0.1	28.37 ± 0.17	33.80 ± 0.60
M6	35.0 ± 1.0	34.5 ± 0.5	1.8 ± 0.2	1.8 ± 0.1	30.68 ± 0.04	34.75 ± 0.05
M7	30.0 ± 0.5	30.5 ± 0.5	1.5 ± 0.1	1.4 ± 0.1	25.59 ± 0.02	29.88 ± 0.57

**Table 10:** Calculated mucoadhesive interaction parameter (ΔG′) of elastic modulus (G′) for mucins/M 2-7 microgels mixtures and their corresponding microgels at 1, 10, and 100 rad/s.

PRD Microgels	M2 (Pa)	(Mucins/M2 Microgel) _mixture_ (Pa)	ΔG′ (Pa)
G′ (1 rad/s)	8377.5	6860.0	−1517.5
G′ (10 rad/s)	9377.5	9520.8	+143.3
G′ (100 rad/s)	9880.0	11,700.0	+1820
**Microgel**	**M3** **(Pa)**	**(Mucins/M3 Microgel) _mixture_** **(Pa)**	**ΔG′** **(Pa)**
G′ (1 rad/s)	9753.3	11,853.3	+2100
G′ (10 rad/s)	10,950.0	14,300.0	+3350
G′ (100 rad/s)	11,533.3	16,233.0	+4699.7
**Microgel**	**M4** **(Pa)**	**(Mucins/M4 Microgel) _mixture_** **(Pa)**	**ΔG′** **(Pa)**
G′ (1 rad/s)	9432.5	9756.7	+324.2
G′ (10 rad/s)	10,925.0	12,766.7	+1841.7
G′ (100 rad/s)	11,700.0	15,266.7	+3566.7
**Microgel**	**M5** **(Pa)**	**(Mucins/M5 Microgel) _mixture_** **(Pa)**	**ΔG′** **(Pa)**
G′ (1 rad/s)	14,700.0	16,600.0	+1900
G′ (10 rad/s)	16,900.0	19,666.7	+2766.7
G′ (100 rad/s)	18,133.3	21,866.7	+3733.4
**Microgel**	**M6** **(Pa)**	**(Mucins/M6 Microgel) _mixture_** **(Pa)**	**ΔG′** **(Pa)**
G′ (1 rad/s)	19,300.0	20,633.3	+1333.3
G′ (10 rad/s)	21,400.0	24,666.7	+3266.7
G′ (100 rad/s)	22,360.0	26,466.7	+4106.7
**Microgel**	**M7** **(Pa)**	**(Mucins/M7 Microgel) _mixture_** **(Pa)**	**ΔG′** **(Pa)**
G′ (1 rad/s)	21,916.7	24,666.7	+2750.0
G′ (10 rad/s)	23,650.0	27,866.7	+4216.7
G′ (100 rad/s)	24,183.3	30,366.7	+6183.4

**Table 11:** Prednisolone (PRD) content in microgels after three months of storage at different conditions.

**PRD Microgels**	**Initial**				**Three Months**	
	**Room Temperature** **(%)**	**30 °C/65% RH** **(%)**	**40 °C/75% RH** **(%)**	**Room Temperature** **(100%)**	**30 °C/65% RH** **(%)**	**40 °C/75% RH** **(%)**
M2	101.5 ± 0.2	103.6 ± 1.1	102.6 ± 2.1	104.4 ± 2.4	100.6 ± 4.5	92.4 ± 2.7
M3	103.2 ± 1.8	101.7 ± 3.8	100.7 ± 1.2	105.4 ± 2.4	104.2 ± 1.0	87.2 ± 2.8
M4	100.7 ± 1.7	100.8 ± 1.9	100.8 ± 1.9	98.0 ± 2.0	100.8 ± 3.2	91.7 ± 6.4
M5	100.4 ± 1.1	100.8 ± 2.2	100.4 ± 1.5	97.4 ± 1.0	96.0 ± 2.5	92.3 ± 1.9
M6	101.0 ± 2.4	101.2 ± 2.2	100.6 ± 2.5	97.4 ± 1.1	97.2 ± 3.3	80.8 ± 4.0
M7	105.2 ± 1.1	101.0 ± 2.1	100.6 ± 2.8	104.4 ± 2.7	98.4 ± 2.6	86.4 ± 4.3

**Table 12:** Values of calculated coefficient of determination (R^2^) and exponent of release mechanism (n) from the release data of M 2-7 microgels using the zero-order, first-order, Higuchi, and Korsmeyer–Peppas models.

PRD Microgels	Zero-Order	First-Order	Higuchi	Korsmeyer–Peppas	
	R^2^	R^2^	R^2^	R^2^	n
M2	0.9986	0.9829	0.9831	0.9987	0.88
M3	0.9996	0.9838	0.9853	0.9997	0.93
M4	0.9986	0.9994	0.9822	0.9994	0.91
M5	0.9961	0.9637	0.9741	0.9946	0.95
M6	0.9982	0.9817	0.9862	0.9988	1.01
M7	0.9956	0.9778	0.9799	0.9973	1.05

## Data Availability

Not applicable.
